# In Silico Knockout Screening of* Plasmodium falciparum* Reactions and Prediction of Novel Essential Reactions by Analysing the Metabolic Network

**DOI:** 10.1155/2018/8985718

**Published:** 2018-03-29

**Authors:** Jelili Oyelade, Itunuoluwa Isewon, Efosa Uwoghiren, Olufemi Aromolaran, Olufunke Oladipupo

**Affiliations:** ^1^Department of Computer and Information Science, Covenant University, Ota, Ogun State, Nigeria; ^2^Covenant University Bioinformatics Research (CUBRe), Covenant University, Ota, Ogun State, Nigeria

## Abstract

Malaria is an infectious disease that affects close to half a million individuals every year and* Plasmodium falciparum* is a major cause of malaria. The treatment of this disease could be done effectively if the essential enzymes of this parasite are specifically targeted. Nevertheless, the development of the parasite in resisting existing drugs now makes discovering new drugs a core responsibility. In this study, a novel computational model that makes the prediction of new and validated antimalarial drug target cheaper, easier, and faster has been developed. We have identified new essential reactions as potential targets for drugs in the metabolic network of the parasite. Among the top seven (7) predicted essential reactions, four (4) have been previously identified in earlier studies with biological evidence and one (1) has been with computational evidence. The results from our study were compared with an extensive list of seventy-seven (77) essential reactions with biological evidence from a previous study. We present a list of thirty-one (31) potential candidates for drug targets in* Plasmodium falciparum* which includes twenty-four (24) new potential candidates for drug targets.

## 1. Introduction

*Plasmodium falciparum*, a leading cause of malaria, has a complex life cycle [1, 2]. The female mosquito of the* Anopheles* genre is responsible for all the transference of malaria from one patient to another [1, 2]. Despite the colossal efforts put in to fight malaria, the disease still affects up to over 200 million people every year with close to half a million dying [1–5]. The* Plasmodium falciparum* lifecycle comprises three (3) important developmental stages: the mosquito stage, the liver stage, and the blood stage [6].* Sporozoites *injected into a host by a mosquito that is infected travel to the liver and begin the hepatic stage of the life cycle of the* Plasmodium* by invading hepatocytes. Here, they get to increase and segregate into schizonts, then comprising numerous hepatic merozoites. All of these merozoites are successively set loose into the blood where the erythrocytic stage is initiated and begins by invading and duplicating inside the red blood cells (RBCs) [1, 7].

Evidences abound to the fact that the parasite is already developing resistance to many front-line antimalaria therapies. Therefore, novel antimalaria cures are in immediate need to combat the drug-resistant malaria parasite [5, 8]. The metabolism of* Plasmodium falciparum (P. f.)* in cells that are infected would be quite a potential source of targets for novel drugs but it is complex and difficult to understand intuitively. In silico methods can handle and take care of this complexity. They also give room for integrative analyses of the cell metabolism [9]. In silico methods play an important role in the identification and prediction of new drugs [10] and facilitate the prospects for the discovery of imminent drug leads [11]. They have been successfully used to predict potential drug targets, alter already existing proteins so as to have an improved stability and functionality, and reduce the search space for drug prediction [5, 8, 12]. The challenge of* P. f.*'s resistance to most identified antimalarial drugs has given rise to the increase of antimalarial drug discovery research [4, 13, 14]. Hence, research on the development of novel drug targets which would serve as an effective solution for malaria treatment is urgently needed [3, 13–15]. Due to upgrades of* P. f.* genome, the reconstruction of the metabolic network is required for a comprehensive understanding of the molecular mechanisms of the organism [8, 16, 17]. Though experimental validation of novel drug target by reaction knockout methods could be accurate, they are time consuming and are a major impediment towards discovery of drug targets [5]. Thus, this study provides a novel computational model that predicts essential reactions and makes the validation of predicted antimalarial drug target cheaper, easier, and faster. It also gives a deeper understanding of the metabolic activities of* P. f*. This study identifies new reactions as potential targets for drugs in the metabolic network of* P. f.* that contributes to its survival in the host and validates predicted drug targets. The computational model used in this paper is an enhancement of the computational model used in our preceding paper [18]. The computational model used enhances the results and enables computational analysis of large dataset.

The malaria parasite metabolic pathways are quite different from those of its human host. This uniqueness can be exploited in the design of therapeutic strategies [19–21]. Metabolic pathways are chains of connected enzymatic reactions that take place inside a cell [22, 23]. They form a different chemical compound by modifying a principal compound which is then passed on to start an alternative pathway, used up or kept by the cell [24]. The representation of a metabolic pathway is generally a graphical network of chemical reactions [24]. The stoichiometry represents the quantifiable relations amid reactants and products in a balanced chemical reaction. Combinations of information from different sources such as genomics, network analysis and simulation, and biochemistry are necessary in the study of a metabolic pathway [25]. More than one metabolic pathway which consists of a chain of reactions that contribute to the synthesis or degradation of the same metabolite makes up a metabolic network. Diverse data sources guide the genome-scale reconstruction of metabolic networks [5]. Marwan et al. [26] regarded metabolic networks as a flow of substance from side to side of biochemical intermediates that are converted into each other. A metabolic network is simply a graphical representation of metabolism [27], characterized by a flow of substance through biochemical intermediates that are interconverted into each other [26]. Metabolic networks are useful tools for deepening our understanding of the metabolism and the role of genes through the evaluation of gene essentiality [28]. Therefore, a metabolic network is simply a diagrammatic illustration of the chemical processes that occur in maintaining the living state of the cells and the organism [27]. KEGG (Kyoto Encyclopaedia of Genes and Genomes) is an integrated primary database resource that consists of 16 main databases for biological interpretation of high-throughput data which are characterized as chemical, systems, genomic, and health information and genome sequences [29]. KEGG is a database resource for comprehending higher order functions and utilities that are comprised in the biological system [30–32]. MetaCyc is a general database consisting of enzymes and metabolic pathways. MetaCyc acts in the capacity of a reference database of small-molecule metabolism which is not redundant and is comprised of metabolic pathways that are experimentally verified and enzyme information selected from the different scientific literature. It makes a unique resource of high quality available for metabolic pathways and enzymes because it comprises only experimentally explained knowledge [33–35]. MetaCyc is one of the major collections of metabolic pathways with over 1700 pathways [34].

### 1.1. Essentiality of a Reaction in a Metabolic Network

**Figure 1:**
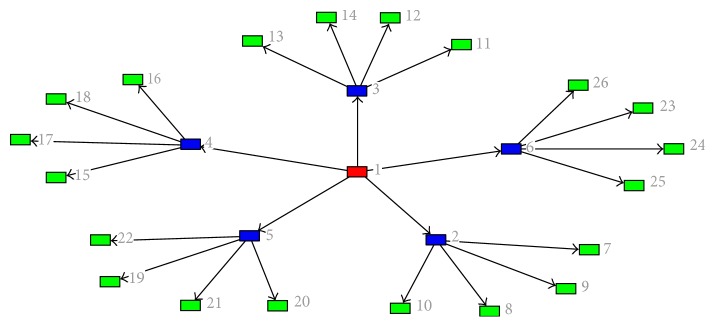
A network tree which paints the essentiality of each node to the survival of the tree [41].

Identifying essential reactions in a metabolic network allows the identification of potential drug targets in the network [36, 37]. Essential reactions are widely recognized as ideal drug target candidates since deleting them could lead to a compromise of integrity of the network [37, 38]. Essential reactions are those reactions of an organism that are thought to be critical for its survival because without them the network cannot function [39]. The predictions of essential reactions experimentally even though largely accurate have a need for substantial time and resources, even for organisms that are well-studied, and they are not at all times practical [5, 37, 40], while the predictions of essential reactions computationally are faster and quite less expensive and they have the capability to decrease the search space for new targets for drugs in a metabolic network which can then be validated experimentally [5, 37]. Deleting just one essential reaction is enough to cause lethality or infertility in the network. In comparison to nonessential reactions, essential reactions are expected to be more preserved in biological evolution [36, 37]. The essentiality of a node in a network is explained in Figure 1 which depicts a network with three different essentiality levels, the red node being the most essential node followed by the blue node in the network and the yellow nodes are the least essential nodes in the network. For example, if the hub node (the red node) is knocked out, it will affect the entire network in the system.

### 1.2. Methods of Detecting Essentiality of Reactions

Flux balance analysis (FBA) is an in silico method used in gaining deepened understanding into the abilities and the metabolic behaviour of a cell [5, 42, 43]. It is an extensively used and deep-rooted method to assess the essential genes of a particular organism. FBA is used extensively in the study of reconstructing the metabolic network of a genome based on mass conservation. Flux balance analysis envisages the complete growth rate of a particular organism or rate of utilization of any particular metabolite by simply calculating how the metabolites flow through the metabolic network [43]. The stoichiometry information of the metabolic network along with the metabolic target functions essential to the cell of interest is also necessitated by FBA [5, 43]. However, there are quite a number of baffling failures of FBA techniques in predicting the essentiality of a gene in a particular organism [44–46]. FBA suffers from incomplete annotation of the proteins in a genome [46]; FBA suffers greatly in defining biologically relevant objective function [46]. The information about the stoichiometry of the reaction pathway is required when using FBA [43]; FBA technique fails to moderately correlate between evolutionary rate and predicted gene dispensability [44, 45]; FBA approach is in need of perfect specification that defines the production of biomass and the nutrition that is available under explicitly given environmental conditions [47]. Minimal Metabolic Behaviours [48] can be seen as mathematical method to approach metabolic pathway analysis; it makes use of the outer description of the steady-state flux cone, which is determined by the sets or number of nonnegativity constraints [48]. When compared to already existing methods, its description is more compacted. It proposes an integrated method to the studying of the metabolic networks [48]. Elementary mode analysis (EMA) is a veritable metabolic pathway tool that considers stoichiometric and thermodynamics when evaluating whether a particular metabolic route or network is feasible and likely for a set of proteins/enzymes [49]. This method is valuable for the purpose of decomposing the intricate metabolic network made up of highly interconnected reactions into uniquely organized pathways. Elementary mode analysis is a tool used to identify the structure of a metabolic network that connects the cellular phenotype to the corresponding genotype. Elementary modes increase rapidly with regard to the network size and because of this the time to compute the network increases largely with respect to the size of the network thereby limiting analysis to pathways and not the entire metabolic network of a genome [50, 51]. EMA is based mainly on the reaction equations stoichiometry and the steady-state conditions of the particular organism [52]. When using metabolic flux analysis (MFA) [53, 54], any change made in the metabolic pathway fluxes is measured. Information like this gives more insights into how the metabolic pathways are being regulated and could likely suggest novel targets for added metabolic engineering of the strains [55]. Metabolic flux analysis (MFA) denotes an influential tool for systems biology research [56]. A major setback of metabolic flux analysis for a lot of biological systems is however that the amount of constraint is often not sufficient to observe all essential intracellular metabolic pathways [55, 57]. In load point and choke point analysis, the number of *k*-shortest paths passing through metabolites and its closest neighbour links is defined as the load point of a particular metabolite in a metabolic network. The usefulness or the importance of a particular metabolite in the metabolic network of an organism is determined by load points and choke points [58–60]. The choke points are ordered by the amount of *k*-shortest paths passing through them. When a choke point in an organism is absent, the organism can rarely survive [59, 60]. Thermodynamics-based Flux Analysis (TFA) is a variant of metabolic flux analysis presented with the capacity of producing thermodynamically feasible flux and metabolite movement profiles on a genome scale [61, 62]. TMFA includes the utilization of an arrangement of straight thermodynamic constraints notwithstanding the mass balance limitations ordinarily utilized as a part of MFA [9, 62]. TMFA produces flux circulations not encompassing any form of thermodynamically infeasible responses or pathways, and in addition to reaction fluxes it makes lots of information about the range of a substance formed or necessary for metabolism activities and the free energy change of reactions available [9, 61, 62]. Metabolic Control Analysis helps in determining quantitatively the level or amount of influence that different enzymes have in the intracellular network on very important flux (or function) [63]. MCA is categorized as a postgenomic device used in comprehending the principles that govern a metabolic network which is disseminated among numerous enzymatic steps [64]. MCA studies provide rational and quantitative criteria to select enzymes for drug target development [65]. The application of Metabolic Control Analysis makes it possible to recognize the group of proteins that necessarily have to be altered to achieve an effective modulation of the intracellular networks of biotechnological or clinical relevance [63].

### 1.3. Reaction Deletion/Perturbation Studies

Perturbation is an approach generally applied to study the conduct and atomic components underlying cellular systems [66]. A perturbation can likewise be focused on the interruption of a specific cell segment, for instance, by deleting reactions or by RNA-intervened knockdown. These two universal types of perturbation are frequently used [66]. Perturbation in a particular pathway happens by interfering with the flow of the signal of a given network which gives knowledge into both their arrangement and their downstream targets. To begin with, with the interference at a specific node in the pathway, the signal cannot be conveyed further. Secondly, every node in the pathway may have its own (immediate or aberrant) commitment to the perturbation impacts, for example, reaction expression changes [67].

In this study, the essentiality of the different reactions was determined. Therefore, a list of indispensable reactions in the* Plasmodium falciparum *metabolic network was identified and proposed as potential drug target for* Plasmodium falciparum*.

## 2. Materials and Methods

### 2.1. Reconstruction of the Metabolic Network

In this study two different resources were considered for the reconstruction of the metabolic network, which is the genome-scale metabolic dataset of the 3D7 strain of* Plasmodium falciparum* which was extracted from the BIOCYC flat file database version 19.5 [68] because of its comprehensiveness and robustness where the dataset contained 894 metabolic reactions and these reactions were catalyzed by a total number of 710 enzymes; also the metabolic dataset of the genome-scale 3D7 strain of* Plasmodium falciparum* from [9] was extracted to fill the gaps in the BIOCYC genome-scale metabolic dataset of which the dataset contained 670 metabolic reactions and these reactions were catalyzed by a total number of 325 enzymes. The raw data used by Chiappino-Pepe includes the protein FASTA files version 11.1 with protein sequences for* P. falciparum* 3D7 from PlasmoDB and the version of KEGG as of July 2014. The BIOCYC identifiers were chosen for this study as the generally accepted means of identification.* Plasmodium falciparum* reactions gotten from Chiappino-Pepe were mapped to BIOCYC reactions via enzymes commission numbers and common name. The reconstructed metabolic network of* Plasmodium falciparum* is made available as SBML file with some reactions considered to be reversible and some considered to be irreversible. In this study, currency metabolites of the 24 currency metabolites outlined by [69] were removed from the reconstructed genome-scale metabolic dataset.

### 2.2. In Silico Knockout Analysis

Once the metabolic network was reconstructed, a* Plasmodium falciparum* metabolic network was created leading us to perform an in silico knockout experiment and analysis on the metabolic network enabling us to analyse the network for the essentiality and perturbation of the knocked-out reactions and we also moved further to determine the perturbations and essentiality of all reactions in the network. When a transition (reaction) is knocked out, all reactions that have a corresponding reactant or product of the knocked-out reaction are equally knocked out, helping to ascertain the effect of that reaction to the network. Single knockout analyses were performed and our results were outputted in  .txt formats to list the reactions that were affected after knocking out a specific reaction.

### 2.3. The Algorithm

In the network a reaction is knocked out to determine the dependent reaction on the knocked-out reaction. The procedure for determining which reaction is connected to the knockout is outlined in the following steps.

**Step 1 .**

Start.

**Step 2 .**

Get the SBML file.

**Step 3 .**

Extract all reactions in the file.

**Step 4 .**

Extract all products and reactants related to the various reactions.

**Step 5 .**

Identify reaction to knockout initially.

**Step 6 .**

Get other reactions that are linked to the identified reaction which can be knocked out.

**Step 7 .**

Determine if the other reactions are linked to other reactions.

**Step 8 .**

If true, do not knock out the reaction; else knock out the reaction.

**Step 9 .**

Repeat Steps 6–8 for the resulting reactions.

**Step 10 .**

If there are no other reactions to be knocked out, attach the resulting reactions to the initial reaction.

**Step 11 .**

Assign “knocked-out reactions” as the list of resulting reactions attached to the initial knocked-out reaction.

**Step 12 .**

Repeat Steps 5–11 to get all results for every reaction in the network.

**Step 13 .**

Determine the essentiality of each reaction by comparing the knocked-out items to the total network.

**Step 14 .**

Determine the most essential reactions by comparing the results of Step 13.

**Step 15 .**

Determine the least essential reactions by comparing the results of Step 13.

**Step 16 .**

Extract result to a spreadsheet file.

**Step 17 .**

Make visualisation of the result in the spreadsheet file.

**Step 18 .**

Stop.

All above steps are performed for all reactions in the network enabling this study to predict the essentiality of all reactions in the network and exporting them in hierarchical order.

(1) $$\begin{array}{c} \text{If }R1=SR\\ Kc\;\forall R\\ \text{If }R\left(\;Re\;\right)=R1\left(\;Pr\;\right)\\ End\\ \text{IF }R1=SR\\ Kc\;\forall R\\ \text{If }R\left(\;Pr\;\right)=R1\left(\;Re\;\right)\\ End\\ \text{IF }R1\left(\;Re\;\right)=R\left(\;Pr\;\right)\\ DKc\;\;R\\ \text{End}\\ \text{IF }R1\left(\;Pr\;\right)=R\left(\;Re\;\right)\\ DKc\;\;R\\ \text{End} \end{array}$$

R represents reaction, SR represents selected reaction, Re represents reactant, Pr represents product, Kc means knockout, and DKc means no knockout.

In this study reconstructed network, we determined the essentiality of every reaction in the network and proposed some essential reactions and validated the essentiality of previous proposed reactions in literature. The essentiality of all reactions is saved in a  .txt file for easy access. The formula for determining the essentiality of every reaction in the network is outlined in the following steps:

(2) ri=r1+r2+r3+⋯+rn

(3) Er=∑kri∑Nri%.

*ri* represents the list of reactions, *E*(*r*) represents the essentiality of a reaction in the reconstructed metabolic network, *k*(*ri*) represents the knocked-out reactions in the reconstructed metabolic network, *N*(*ri*) represents a reaction in the network, ∑*k*(*ri*) represents the summation of the knocked-out reactions in the reconstructed metabolic network, and ∑*N*(*ri*) represents the summation of all reactions in the network.

### 2.4. The Gold Standard

An extensive list of 77 essential reactions in* Plasmodium falciparum* which mostly have been predicted in several literatures to be druggable was considered when testing and validating our model and these 77 essential reactions are given in the supplement (supplementary Table S1). A large percentage of this gold standard was considered in our network and validated by our model as essential. Our network was constructed as a directed-bipartite graph with two different types of nodes.

## 3. Results and Discussion

### 3.1. Results

Computationally predicted essential reactions from six different literatures were compared with our method. Ten reactions that were common to over 80% of all literature considered in this study were identified and validated to be essential by our method, thereby confirming our method as valuable computational technique for validation of predicted drug target as given in Table 1, respectively. The network of* Plasmodium falciparum *used in our study was analysed and each reaction in the network was knocked out and the essentiality of each reaction in the network was determined. The top seven (7) predicted most essential reactions based on our method in the network are represented in Table 2, four (4) of which were identified to be found in the gold standards including superoxide dismutase, 3-phosphoshikimate 1-carboxyvinyltransferase, 5-O-(1-carboxyvinyl)-3-phosphoshikimate phosphate-lyase, and adenosylhomocysteinase and one (1) was already predicted as essential computationally which is methionine adenosyltransferase. The reactions are well represented by BIOCYC database reaction unique identification number. The reactions that are represented boldly are reactions that are represented in gold standards.

#### 3.1.1. Knocked-Out Reactions for the Most Essential Reactions

The reactions that were knocked out from the network when the top seven (7) most essential reactions from our network were knocked out are given in the Supplementary Table S2(a–g). Table S2(a) represents the reactions that were knocked out by reaction SUPEROX-DISMUT-RXN which is responsible for knocking out 94 other reactions in the metabolic network. Table S2(b) represents the reactions that were knocked out by reaction CATAL-RXN of which is responsible for knocking out another 74 reactions in the network. In Table S2(c–g), reactions S-ADENMETSYN-RXN, 2.5.1.19-RXN, CHORISMATE-SYNTHASE-RXN, SHIKIMATE-KINASE-RXN, and ADENOSYLHOMOCYSTEINASE-RXN, respectively, were responsible for knocking out another 49, 48, 48, 48, and 39 reactions in the metabolic network. Table S2(d) represents the reactions that were knocked out by reaction 2.5.1.19-RXN which is responsible for knocking out another 48 reactions in the metabolic network used.

The predicted essential reactions based on the analysis of our network are given in the Supplementary Table S3 and Figures S1 and S2. Table S3 lists all reactions that are seemingly essential to the network used in this study and their essentiality level where these reactions were compared with gold standards and computationally predicted reactions. This study finally presents a polished list of 31 potential candidates for drug targets in* Plasmodium falciparum *which includes 24 new potential candidates for drug targets of which 9 are orphans and 7 potential candidates for drug target which has been predicted computationally in literature of which 1 is an orphan. The reactions are presented in Table 3.

### 3.2. Discussion

This study established a novel method that performed an analysis on the genome-scale metabolic network of* Plasmodium falciparum *and identifies reactions in the network that are essential to the survival of the network according to its essentiality. These essential reactions are predicted as potential drug targets for* Plasmodium falciparum; *the essentiality of the reaction in the network is listed according to its effect on the network when knocked out (the amount of reactions knocked out when the parent reaction is knocked out). This study identifies that there are over 200 essential reactions in the network of which, among the top 7 predicted most essential reactions, 4 were identified to be found in the gold standard which includes superoxide dismutase, 3-phosphoshikimate 1-carboxyvinyltransferase, 5-O-(1-carboxyvinyl)-3-phosphoshikimate phosphate-lyase, and adenosylhomocysteinase and 1 was already predicted as essential computationally which is methionine adenosyltransferase. The result of our study was compared with an extensive list of 77 essential reactions with biological evidence. We finally present a polished list of 31 including 24 new potential candidates for drug targets of which 9 are orphans and 7 potential candidates for drug target which has been predicted computationally in literature of which 1 is an orphan. This model also helps to improve the understanding of the biological processes within this network and any other metabolic network. It would be quite exciting to further our research by confirming our in silico predictions experimentally and also test if our essential reactions can be successfully targeted without collateral partial or complete targeting of the corresponding human reactions. Potential candidates for drug targets for* Plasmodium falciparum *already biologically proven were disregarded from our list as our method validated a number of them. The method developed could handle multiple knockouts but we plan to do this when we have some amount of drug combination to validate our method. The method used in this study is capable of predicting essential reaction in any other organisms of a robust genome-scale metabolic network.

## 4. Conclusion

The dominance of malaria in resistance to identified antimalarial drugs in current circulation has given rise to the increase of antimalarial drug discovery research. Hence, researches on the development of novel drug targets which would serve as effective solutions for malaria treatment are urgently needed.

In this study, a novel computational model was constructed which makes the validation of predicted antimalarial drug target cheaper, easier, and faster as well as the validation of* P. f. *metabolic reactions under different growth conditions and perturbations. We have been able to identify new essential reactions as possible targets for drugs in the metabolic network of* P. f. *that contributes to its survival in the host and validate predicted drug targets. The computational model used in this study enhances and enables computational analysis of large dataset.

Generally, the results from this study make a deep understanding of the metabolism of* P. f. *available and provide guidance to experimental studies helping to develop a better description of* P. falciparum* metabolism and to identify antimalarial drug targets.

## Figures and Tables

**Table 1:** Results using our method to validate existing potential drug target predicted computationally [18].

S/N	Reaction	EC number	Yeh et al. [17]	Fatumo et al. [47]	Huthmacher et al. [5]	Plata et al. [8]	Bazzani et al. [70]	Chiappino-Pepe et al. [9]	This study
(1)	DIHYDROFOLATEREDUCT-RXN	1.5.1.3	Yes	Yes	Yes	Yes	Yes	Yes	Yes
(2)	THYMIDYLATESYN-RXN	2.1.1.45	Yes	Yes	Yes	Yes	Yes	Yes	Yes
(3)	H2PTEROATESYNTH-RXN	2.5.1.15	Yes	Yes	Yes	Yes	No	No	Yes
(4)	IMP-DEHYDROG-RXN	1.1.1.205	Yes	Yes	Yes	No	Yes	No	Yes
(5)	SUPEROX-DISMUT-RXN	1.15.1.1	Yes	Yes	Yes	No	Yes	No	Yes
(6)	ADENOSYLHOMOCYSTEINASE-RXN	3.3.11	Yes	Yes	Yes	No	Yes	No	Yes
(7)	OROTPDECARB-RXN	4.1.1.23	Yes	Yes	Yes	Yes	Yes	Yes	Yes
(8)	SAMDECARB-RXN	4.1.1.50	Yes	Yes	Yes	Yes	Yes	No	Yes
(9)	PORPHOBILSYNTH-RXN	4.2.1.24	Yes	Yes	Yes	Yes	Yes	No	Yes
(10)	GLUTCYSLIG-RXN	6.3.2.2	Yes	Yes	Yes	No	Yes	No	Yes

**Table 2:** The top seven predicted most essential reactions from this study.

S/N	Reaction	Gene ID	Common name	EC number	Number of affected reactions	Essentiality
(1)	**SUPEROX-DISMUT-RXN**	PFF1130C, PF08_0071	Superoxide dismutase	1.15.1.1	94	1.27027027
(2)	CATAL-RXN		Catalase	1.11.1.6	74	1
(3)	***S-ADENMETSYN-RXN***	PFI1090W	Methionine adenosyltransferase	2.5.1.6	49	0.662162162
(4)	**2.5.1.19-RXN**		3-Phosphoshikimate 1-carboxyvinyltransferase	2.5.1.19	48	0.648648649
(5)	**CHORISMATE-SYNTHASE-RXN**	PFF1105C	5-O-(1-Carboxyvinyl)-3-phosphoshikimate phosphate-lyase	4.2.3.5	48	0.648648649
(6)	SHIKIMATE-KINASE-RXN		Shikimate kinase	2.7.1.71	48	0.648648649
(7)	**ADENOSYLHOMOCYSTEINASE-RXN**	PFE1050W	Adenosylhomocysteinase	3.3.1.1	39	0.527027027

**Table 3:** 31 potential reactions for drug targets in *Plasmodium falciparum *from this study.

S/N	Reaction	Gene ID	Common name	EC number
(1)	CATAL-RXN	ORPHAN	Catalase	1.11.1.6
(2)	RXN-1321	ORPHAN	Linoleate 13S-lipoxygenase	1.13.11.12
(3)	12-OXOPHYTODIENOATE-REDUCTASE-RXN	ORPHAN	12-Oxophytodienoate reductase	1.3.1.42
(4)	***RXN0-1461***	PF11_0436	Coproporphyrinogen oxidase	1.3.3.3
(5)	AICARTRANSFORM-RXN	ORPHAN	Phosphoribosylaminoimidazolecarboxamide formyltransferase	2.1.2.3
(6)	**METHYLACETOACETYLCOATHIOL-RXN**	PF14_0484	Acetyl-CoA C-acetyltransferase	2.3.1.9
(7)	***S-ADENMETSYN-RXN***	PFI1090W	Methionine adenosyltransferase	2.5.1.6
(8)	**OHMETHYLBILANESYN-RXN**	PFL0480W	Hydroxymethylbilane synthase	2.5.1.61
(9)	**ACETYLORNTRANSAM-RXN**	PFF0435W	Acetylornithine transaminase	2.6.1.11
(10)	**2.6.1.18-RXN**	PFF0435W	*β*-Alanine—pyruvate transaminase	2.6.1.18
(11)	***PABASYN-RXN***	PFI1100W	Aminodeoxychorismate synthase	2.6.1.85
(12)	SHIKIMATE-KINASE-RXN	ORPHAN	Shikimate kinase	2.7.1.71
(13)	UDPKIN-RXN	ORPHAN	Nucleoside-diphosphate kinase	2.7.4.6
(14)	***DTMPKI-RXN***	PFL2465C	dTMP kinase	2.7.4.9
(15)	**H2PTERIDINEPYROPHOSPHOKIN-RXN**	PF08_0095	2-Amino-4-hydroxy-6-hydroxymethyldihydropteridine diphosphokinase	2.7.6.3
(16)	***NICONUCADENYLYLTRAN-RXN***	PF13_0159	Nicotinate-nucleotide adenylyltransferase	2.7.7.18
(17)	**NAG1P-URIDYLTRANS-RXN**	MAL13P1.218	UDP-N-acetylglucosamine diphosphorylase	2.7.7.23
(18)	**GLUC1PURIDYLTRANS-RXN**	MAL13P1.218	UTP—glucose-1-phosphate uridylyltransferase	2.7.7.9
(19)	**2.7.8.15-RXN**	PFC0935C	UDP-N-acetylglucosamine—dolichyl-phosphate N-acetylglucosaminephosphotransferase	2.7.8.15
(20)	**RXN-6384**	PFL1940W	3-Hydroxyisobutyryl-CoA hydrolase	3.1.2.4
(21)	**3-HYDROXYISOBUTYRYL-COA-HYDROLASE-RXN**	PFL1940W	3-Hydroxyisobutyryl-CoA hydrolase	3.1.2.4
(22)	IMPCYCLOHYDROLASE-RXN	ORPHAN	IMP cyclohydrolase	3.5.4.10
(23)	**GTP-CYCLOHYDRO-I-RXN**	PFL1155W	GTP cyclohydrolase I	3.5.4.16
(24)	***UROGENDECARBOX-RXN***	PFF0360W	Uroporphyrinogen decarboxylase	4.1.1.37
(25)	**ADCLY-RXN**	PFI1100W	Aminodeoxychorismate lyase	4.1.3.38
(26)	**PORPHOBILSYNTH-RXN**	PF14_0381	Porphobilinogen synthase	4.2.1.24
(27)	*UROGENIIISYN-RXN*	ORPHAN	Uroporphyrinogen-III synthase	4.2.1.75
(28)	RXN1F-19	ORPHAN	Hydroperoxide dehydratase	4.2.1.92
(29)	ALLENE-OXIDE-CYCLASE-RXN	ORPHAN	Allene-oxide cyclase	5.3.99.6
(30)	**TRYPTOPHAN--TRNA-LIGASE-RXN**	PF13_0205, PFL2485C	Tryptophan—tRNA ligase	6.1.1.2
(31)	**HISTIDINE--TRNA-LIGASE-RXN**	PF14_0428	Histidine—tRNA ligase	6.1.1.21

## Data Availability

The datasets used in this study are available in http://bioinformatics.ai.sri.com/ecocyc/dist/flatfiles-52983746/.

## Supplementary Materials

Supplementary Materials
* Appendix A*. Table S1: the gold standard. This consists of reactions which are biologically proven to be targets of already accepted/tested drugs. Table S2: knocked-out reactions for reactions a–g.* Appendix B*. Table S3: all predicted essential reactions in the network used in this study. The predicted essential reactions based on our analysis in the network are presented in this table, most of which were identified to be found in the gold standards or have been predicted to be essential to the network in previous study. Figure S1: graph to show number of reactions considered and essentiality of reactions. Figure S2: graph to represent essentiality of reaction.
