# Supplementary material for: In Silico Knockout Screening of Plasmodium falciparum Reactions and Prediction of Novel Essential Reactions by Analysing the Metabolic Network
Source: Biomed Res Int. 2018 Mar 29;2018:8985718. doi: 10.1155/2018/8985718 (PMC5896307; doi:10.1155/2018/8985718)
Supplement: Supplementary Materials — Appendix A. Table S1: the gold standard. This consists of reactions which are biologically proven to be targets of already accepted/tested drugs. Table S2: knocked-out reactions for reactions a–g. Appendix B. Table S3: all predicted essential reactions in the network used in this study. The predicted essential reactions based on our analysis in the network are presented in this table, most of which were identified to be found in the gold standards or have been predicted to be essential to the network in previous study. Figure S1: graph to show number of reactions considered and essentiality of reactions. Figure S2: graph to represent essentiality of reaction. [file 8985718.f1.pdf]

Appendix A. Supplementary material

Table S1: The gold standard

Enzyme Commission No.	Reaction	Reference
None	histone deacetylase	[71], [72]
1.1.1.205	IMP dehydrogenase	[73]
1.1.1.267	1-deoxy-D-xylulose 5-phosphate reductoisomerase	[74]–[76]
1.1.1.27	lactate dehydrogenase	[77], [78]
1.10.2.2	mitochondrial ubiquinol-cytochrome C reductase	[79]
1.15.1.1	superoxide dismutase	[80]
1.17.1.2	4-hydroxy-3-methylbut-2-enyl diphosphate reductase	[81]
1.17.4.1	Ribonucleoside-diphosphate reductase	[82], [83]
1.2.4.4	3-Methyl-2-oxobutanoate dehydrogenase (lipoamide)	[84]
1.3.1.9	enoyl-ACP-reductase	[85], [86]
1.3.3.1	Dihydroorotate oxidase	[87]–[93]
1.3.3.4	Protoporphyrinogen oxidase	[94], [95]
1.3.99.1	Succinate dehydrogenase	[96]
1.5.1.3	Dihydrofolate reductase	[97]–[99]
1.5.1.5, 3.5.4.9	Methylenetetrahydrofolate dehydrogenase (NADP+), methenyltetrahydrofolate cyclohydrolase	[99]
1.6.5.3	NADH dehydrogenase (ubiquinone)	[100]
1.6.99.3	NADH dehydrogenase	[101], [102]
1.8.1.7	glutathione reductase	[103]–[106]
1.8.1.9	thioredoxin reductase	[107]
2.1.1.100	Protein-S-isoprenylcysteine-O-methyltransferase	[108], [109]
2.1.1.103	phosphoethanolamine N-methyltransferase	[110]
2.1.1.45	Thymidylate synthase	[98], [111]
2.1.1.64	3-Demethylubiquinone-9,3-O-methyltransferase	[99]
2.1.3.2	aspartate carbamoyltransferase	[112]
2.2.1.7	1-deoxy-D-xylulose-5-phosphate synthase	[74], [113]
2.3.1.15	Glycerol-3-phosphate-O-acyltransferase	[114]–[116]
2.3.1.24	sphingosine N-acyltransferase	[117]
2.3.1.37	5-aminolevulinate synthase	[118], [119]
2.3.1.41	3-Oxoacyl-[acyl-carrier protein] synthase	[85], [120]–[122]
2.3.1.50	serine C-palmitoyltransferase	[117]
2.4.2.1	Purine-nucleoside phosphorylase	[123], [124]
2.4.2.10	orotate phosphoribosyltransferase	[125], [126]

2.4.2.8	Hypoxanthine phosphoribosyltransferase	[127], [128]
2.5.1.10, 2.5.1.1	Geranyltranstransferase, dimethylallyltranstransferase	[129]
2.5.1.15	Dihydropteroate synthase	[130]
2.5.1.16	Spermidine synthase	[131]
2.5.1.18	Glutathione transferase	[132]–[135]
2.5.1.19	3-Phosphoshikimate 1-carboxyvinyltransferase	[136]
2.5.1.21	Farnesyl-diphosphate farnesyltransferase	[137]
2.5.1.46	deoxyhypusine synthase	[138]
2.5.1.58	protein farnesyltransferase	[139]
2.7.1.32	Choline kinase	[140], [141]
2.7.8.3	sphingomyelin synthase/phosphatidic acid phosphatase	[117]
3.1.1.31, 1.1.1.49	6-phosphogluconolactonase, glucose-6-phosphate dehydrogenase	[142], [143]
3.1.1.5	Lysophospholipase	[144]
3.1.3.56	Inositol-1,4,5-trisphosphate 5-phosphatase	[145]
3.1.4.12	Sphingomyelin phosphodiesterase	[146]
3.1.4.17	3′,5′-Cyclic-nucleotide phosphodiesterase	[147], [148]
3.3.1.1	S-adenosyl-l-homocysteine hydrolase	[149]–[151]
3.5.2.3	Dihydroorotase	[152], [153]
3.5.4.4	Adenosine deaminase	[154], [155]
3.6.1.17	Bis(5′-nucleosyl)-tetraphosphatase (asymmetrical)	[156]
4.1.1.17	Ornithine decarboxylase	[157]–[159]
4.1.1.23	Orotidine-5′-phosphate decarboxylase	[126], [160]–[162]
4.1.1.50	Adenosylmethionine decarboxylase	[158], [159], [163], [164]
4.1.2.13	Fructose-bisphosphate aldolase	[165]
4.2.1.1	carbonate dehydratase	[166]
4.2.1.11	Phosphopyruvate hydratase / enolase	[167]
4.2.1.24	Delta-aminolevulinic acid dehydratase	[95], [168]
4.2.1.58 – 61	3-hydroxyacyl-ACP dehydratase	[85], [169]
4.2.3.5	Chorismate synthase	[90]
4.3.2.2	adenylosuccinate lyase	[170]
4.4.1.5	Lactoylglutathione lyase	[171], [172]
4.6.1.12	2C-methyl-D-erythritol 2,4-cyclodiphosphate synthase	[173]
5.3.1.1	triosephosphate isomerase	[174]
5.99.1.2	topoisomerase I	[175]
5.99.1.3	topoisomerase II	[176]–[178]
6.1.1.3	Threonine-tRNA ligase	[179]

6.1.1.7	Alanine-tRNA ligase	[180]
6.3.2.12	dihydrofolate synthase	[181]
6.3.2.17	folylpolyglutamate synthase	[181]
6.3.2.2	Gamma-glutamylcysteine synthetase	[105], [182], [183]
6.3.5.5	Carbamoyl-phosphate synthase (glutamine-hydrolysing)	[184]
6.3.4.4	adenylosuccinate synthase	[185]
6.3.5.2	GMP synthase	[186]
6.3.5.5	carbamoyl phosphate synthetase	[184]
6.4.1.2	Acetyl-CoA carboxylase	[120]

Table S2: Knocked out reactions for reactions a-g

	Reaction	Knocked Out Reaction
a.	SUPEROX-DISMUT-RXN	'RXN-10696', 'RXN-11026', 'PROTOPORGENOXI-RXN', 'RXN-3761', 'THIOL-OXIDASE-RXN', 'L-ASPARTATE-OXID-RXN', 'RXN66-2', 'GLUTATHIONE-PEROXIDASE-RXN', 'RXN-7650', 'DIHYDROOROTOX-RXN', 'RXN-10707', 'RXN-10706', 'RXN-744', 'PNPOXI-RXN', 'RXN3O-1380', 'RXN0-1461', 'CYSTEINE-DIOXYGENASE-RXN', 'SARCOX-RXN', 'RXN-7141', 'PMPOXI-RXN', 'RXN-7686', 'ACYL-COA-OXIDASE-RXN-BUTYRYL-COA/OXYGEN-MOLECULE', 'RXN-1541', 'RXN66-1', 'CATAL-RXN', 'RXN0-3921', 'RXN-1321', 'RXN-10697', 'RXN-10695', 'RXN-3645', 'RXN-5502', 'QUINOLINATE-SYNTHA-RXN', 'RXN-10705', 'RXN-10700', 'RXN-10704', 'RXN-10699', 'PNKIN-RXN', 'PROTOPORGENOXI-RXN', 'UROGENDECARBOX-RXN', '3-SULFINOALANINE-AMINOTRANSFERASE-RXN', 'N-CARBAMOYLSARCOSINE-AMIDASE-RXN', 'R10-RXN', 'RXN-2881', 'CREATINASE-RXN', 'RXN-7224', 'RXN-7226', 'RXN-7779', 'RXN-7142', 'PYRAMKIN-RXN', 'RXN-3303', 'RXN-5501', 'RXN0-3922-GAMMA-GLUTAMYL-GAMMA-AMINOBUTYRALDEH/NAD/WATER', 'RXN0-3922-GAMMA-GLUTAMYL-GAMMA-AMINOBUTYRALDEH/NADP/WATER', 'RXN0-3901', 'RXN1F-19', 'L-ASPARTATE-OXID-RXN', 'QUINOPRIBOTRANS-RXN', 'RXN-10707', 'RXN-10703', 'RXN-10702', 'RXN-10707', 'RXN-10706', 'RXN-10698', 'RXN0-1461', 'UROGENIIISYN-RXN', 'CYSTEINE-DIOXYGENASE-RXN', '3-SULFINYL-PYRUVATE-SPON-RXN', '3.5.2.14-RXN', 'R12-RXN', 'CREATININASE-RXN', 'RXN-7225', 'RXN-7227', 'RXN-7141', 'RXN-3645', 'RXN-7781', 'RXN-7143', 'RXN-7780', 'RXN-3642', 'RXN-1321', 'ALLENE-OXIDE-CYCLASE-RXN', 'RXN-10705', 'RXN-10701', 'OHMETHYLBILANESYN-RXN', 'UROGENDECARBOX-RXN', 'N-CARBAMOYLSARCOSINE-AMIDASE-RXN', 'CREATININE-DEAMINASE-RXN', 'RXN1F-19', '12-OXOPHYTODIENOATE-REDUCTASE-RXN', 'PORPHOBILSYNTH-RXN', 'UROGENIIISYN-RXN', '5-AMINOLEVULINIC-ACID-SYNTHASE-RXN', 'GSAAMINOTRANS-RXN', 'OHMETHYLBILANESYN-RXN', 'GLUTRNAREDUCT-RXN'
b.	CATAL-RXN	'CYSTEINE-DIOXYGENASE-RXN', 'RXN-7650', 'RXN66-2', 'RXN-3761', 'RXN-7141', 'RXN-1321', 'RXN-1541', 'RXN-744', 'RXN3O-1380', 'THIOL-OXIDASE-RXN', 'RXN-7686', 'RXN0-1461', '3-SULFINOALANINE-AMINOTRANSFERASE-RXN', 'RXN-3645', 'RXN-5502', 'RXN-7142', 'RXN-7226', 'RXN-7779', 'RXN-7224', 'RXN1F-19', 'RXN-3303', 'RXN-5501', 'UROGENDECARBOX-RXN', 'PROTOPORGENOXI-RXN', 'CYSTEINE-DIOXYGENASE-RXN', '3-SULFINYL-PYRUVATE-SPON-RXN', 'RXN-7227', 'RXN-7225', 'ALLENE-OXIDE-CYCLASE-RXN', 'RXN-1321', 'RXN-7780', 'RXN-3645', 'RXN-7143', 'RXN-3642', 'RXN-7781', 'RXN-7141', 'UROGENIIISYN-RXN', 'RXN0-1461', 'RXN1F-19', '12-OXOPHYTODIENOATE-REDUCTASE-RXN', 'UROGENDECARBOX-RXN', 'OHMETHYLBILANESYN-RXN', 'ALLENE-OXIDE-CYCLASE-RXN', 'RXN-10695', 'RXN-744', 'UROGENIIISYN-RXN', 'PORPHOBILSYNTH-RXN', 'RXN-10696', '5-AMINOLEVULINIC-ACID-SYNTHASE-RXN', 'GSAAMINOTRANS-RXN', 'OHMETHYLBILANESYN-RXN', 'RXN-10697', 'RXN-10695', 'GLUTRNAREDUCT-RXN', 'RXN-10696', 'RXN-10698', 'RXN-10699', 'RXN-10697', 'RXN-10698', 'RXN-10706', 'RXN-10699', 'RXN-10704', 'RXN-10706', 'RXN-10702', 'RXN-10704', 'RXN-10700', 'RXN-10702', 'RXN-10707', 'RXN-10705', 'RXN-10700', 'RXN-10703', 'RXN-10707', 'RXN-10705', 'RXN-10701'
c.	S-ADENMETSYN-RXN	'RXN-5501', 'RXN-5762', 'ADOMET-DMK-METHYLTRANSFER-RXN', 'DIPHTHINE-SYNTHASE-RXN', 'SAMDECARB-RXN', 'RXN-5763', 'RXN1F-28', 'RXN-3645', '2.1.1.71-RXN', '2-OCTAPRENYL-6-OHPHENOL-METHY-RXN', '2.1.1.5-RXN', '2.1.1.62-RXN', '2.1.1.137-RXN', 'RXN-5643', 'HOMOCYSMET-RXN', '2.1.1.77-RXN', 'RXN-5642', 'FORMYLMETHIONINE-DEFORMYLASE-RXN', 'RXN-5761', '2.1.1.138-RXN', 'RXN-3642', '2.1.1.103-RXN', '2-OCTAPRENYL-METHOXY-BENZOQ-METH-RXN', 'PYRIMSYN1-RXN', 'HOMOCYSMETB12-RXN', 'RXN-5502', 'DMK-RXN',

		'MALATE-DEHYDROGENASE-ACCEPTOR-RXN-MAL/DEMETHYLMENAQUINONE', 'DLACTDEHYDROGFAD-RXN-D-LACTATE/DEMETHYLMENAQUINONE', 'RXN0-5260-DEMETHYLMENAQUINONE/GLYCEROL-3P', 'SPERMINE-SYNTHASE-RXN', 'RXN0-5217', 'SPERMIDINESYN-RXN', 'RXN-745', 'RXN4FS-3', 'RXN-5643', '2.7.7.57-RXN', 'RXN-5642', 'RXN-9311', 'LYSDECARBOX-RXN', 'RXN-744', 'RXN-10708', 'RXN1F-28', 'NAPHTHOATE-SYN-RXN', 'DMK-RXN', 'RXN-9311', 'O-SUCCINYLBENZOATE-COA-LIG-RXN', 'NAPHTHOATE-SYN-RXN', 'O-SUCCINYLBENZOATE-COA-SYN-RXN'
d.	2.5.1.19-RXN	'CHORISMATE-SYNTHASE-RXN', 'SHIKIMATE-KINASE-RXN', 'CHORPYRLY-RXN', 'CHORISMATEMUT-RXN', 'ISOCHORSYN-RXN', 'PABASYN-RXN', 'ANTHRANSYN-RXN', '2.5.1.19-RXN', 'SHIKIMATE-5-DEHYDROGENASE-RXN', '2.5.1.19-RXN', 'RXN-7968-SHIKIMATE/NAD', '4OHBENZOATE-OCTAPRENYLTRANSFER-RXN', '2.5.1.39-RXN', 'PREPHENATEDEHYDRAT-RXN', 'PREPHENATEDEHYDROG-RXN', 'ISOCHORMAT-RXN', 'ADCLY-RXN', 'PRTRANS-RXN', '3-OCTAPRENYL-4-OHBENZOATE-DECARBOX-RXN', 'TYRAMINOTRANS-RXN', 'ORNITHINE--OXO-ACID-AMINOTRANSFERASE-RXN-L-ORNITHINE/P-HYDROXY-PHENYLPYRUVATE', 'DHBDEHYD-RXN', 'ISOCHORSYN-RXN', 'PABASYN-RXN', 'RXN-6142', 'H2PTEROATESYNTH-RXN', 'PRAISOM-RXN', 'ANTHRANSYN-RXN', 'ISOCHORMAT-RXN', 'ENTMULTI-RXN', 'DIHYDROFOLATESYNTH-RXN', 'H2PTERIDINEPYROPHOSPHOKIN-RXN', 'IGPSYN-RXN', 'PRTRANS-RXN', 'H2NEOPTERINALDOL-RXN', 'H2PTEROATESYNTH-RXN', 'PRAISOM-RXN', 'RXN0-2381', 'DIHYDRONEOPTERIN-MONO-P-DEPHOS-RXN', 'H2PTERIDINEPYROPHOSPHOKIN-RXN', 'IGPSYN-RXN', 'RXN0-2382', 'H2NEOPTERINALDOL-RXN', 'H2NEOPTERINP3PYROPHOSPHOHYDRO-RXN', 'TRYPTOPHAN--TRNA-LIGASE-RXN', 'RXN0-2381', 'DIHYDRONEOPTERIN-MONO-P-DEPHOS-RXN', 'GTP-CYCLOHYDRO-I-RXN'
e.	CHORISMATE-SYNTHASE-RXN	'CHORPYRLY-RXN', '2.5.1.19-RXN', 'CHORISMATEMUT-RXN', 'ISOCHORSYN-RXN', 'PABASYN-RXN', 'ANTHRANSYN-RXN', '4OHBENZOATE-OCTAPRENYLTRANSFER-RXN', '2.5.1.39-RXN', 'SHIKIMATE-KINASE-RXN', 'CHORISMATE-SYNTHASE-RXN', 'PREPHENATEDEHYDRAT-RXN', 'PREPHENATEDEHYDROG-RXN', 'ISOCHORMAT-RXN', 'ADCLY-RXN', 'PRTRANS-RXN', 'TYRAMINOTRANS-RXN', 'ORNITHINE--OXO-ACID-AMINOTRANSFERASE-RXN-L-ORNITHINE/P-HYDROXY-PHENYLPYRUVATE', 'ISOCHORSYN-RXN', 'DHBDEHYD-RXN', 'PABASYN-RXN', 'RXN-6142', 'H2PTEROATESYNTH-RXN', 'PRAISOM-RXN', 'ANTHRANSYN-RXN', 'ISOCHORMAT-RXN', 'ENTMULTI-RXN', 'DIHYDROFOLATESYNTH-RXN', 'H2PTERIDINEPYROPHOSPHOKIN-RXN', 'IGPSYN-RXN', 'PRTRANS-RXN', '3-OCTAPRENYL-4-OHBENZOATE-DECARBOX-RXN', '2.5.1.19-RXN', 'SHIKIMATE-5-DEHYDROGENASE-RXN', 'RXN-7968-SHIKIMATE/NAD', 'H2NEOPTERINALDOL-RXN', 'H2PTEROATESYNTH-RXN', 'PRAISOM-RXN', 'RXN0-2381', 'H2PTERIDINEPYROPHOSPHOKIN-RXN', 'DIHYDRONEOPTERIN-MONO-P-DEPHOS-RXN', 'IGPSYN-RXN', 'RXN0-2382', 'H2NEOPTERINALDOL-RXN', 'H2NEOPTERINP3PYROPHOSPHOHYDRO-RXN', 'TRYPTOPHAN--TRNA-LIGASE-RXN', 'RXN0-2381', 'GTP-CYCLOHYDRO-I-RXN', 'DIHYDRONEOPTERIN-MONO-P-DEPHOS-RXN'
f.	SHIKIMATE-KINASE-RXN	'RXN-7968-SHIKIMATE/NAD', 'SHIKIMATE-5-DEHYDROGENASE-RXN', '2.5.1.19-RXN', 'SHIKIMATE-KINASE-RXN', 'CHORISMATE-SYNTHASE-RXN', 'ANTHRANSYN-RXN', 'CHORISMATEMUT-RXN', '2.5.1.19-RXN', 'PABASYN-RXN', 'CHORPYRLY-RXN', 'ISOCHORSYN-RXN', 'PREPHENATEDEHYDROG-RXN', 'PREPHENATEDEHYDRAT-RXN', 'ADCLY-RXN', '4OHBENZOATE-OCTAPRENYLTRANSFER-RXN', '2.5.1.39-RXN', 'ISOCHORMAT-RXN', 'ORNITHINE--OXO-ACID-AMINOTRANSFERASE-RXN-L-ORNITHINE/P-HYDROXY-PHENYLPYRUVATE', 'TYRAMINOTRANS-RXN', 'PABASYN-RXN', 'RXN-6142', 'H2PTEROATESYNTH-RXN', '3-OCTAPRENYL-4-OHBENZOATE-DECARBOX-RXN', 'DHBDEHYD-RXN', 'ISOCHORSYN-RXN', 'DIHYDROFOLATESYNTH-RXN', 'H2PTERIDINEPYROPHOSPHOKIN-RXN', 'ENTMULTI-RXN', 'ISOCHORMAT-RXN', 'PRTRANS-RXN', 'H2NEOPTERINALDOL-RXN', 'H2PTEROATESYNTH-RXN', 'ANTHRANSYN-RXN', 'PRAISOM-RXN', 'H2PTERIDINEPYROPHOSPHOKIN-RXN', 'DIHYDRONEOPTERIN-MONO-P-DEPHOS-RXN', 'PRTRANS-RXN', 'IGPSYN-RXN', 'H2NEOPTERINALDOL-RXN', 'H2NEOPTERINP3PYROPHOSPHOHYDRO-RXN', 'PRAISOM-RXN', 'RXN0-2381', 'GTP-CYCLOHYDRO-I-RXN', 'DIHYDRONEOPTERIN-MONO-P-DEPHOS-RXN', 'IGPSYN-RXN', 'RXN0-2382', 'RXN0-2381', 'TRYPTOPHAN--TRNA-LIGASE-RXN'
g.	ADENOSYLHOMOCYSTEINASE-RXN	'RXN-5761', 'RXN-5501', 'ADOMET-DMK-METHYLTRANSFER-RXN', '2.1.1.62-RXN', '2.1.1.103-RXN', 'RXN-5762', '2.1.1.71-RXN', '2-OCTAPRENYL-METHOXY-BENZOQ-METH-RXN', '2.1.1.77-RXN', 'RXN-3642', 'RXN-5642', 'RXN-5643', 'RXN1F-28', '2.1.1.137-RXN', 'RXN-5763', 'DIPHTHINE-SYNTHASE-RXN', 'RXN-3645', '2-OCTAPRENYL-6-OHPHENOL-METHY-RXN', '2.1.1.138-RXN', 'RXN-5502', 'MALATE-DEHYDROGENASE-ACCEPTOR-RXN-MAL/DEMETHYLMENAQUINONE', 'RXN0-5260-DEMETHYLMENAQUINONE/GLYCEROL-3P', 'DMK-RXN', 'DLACTDEHYDROGFAD-RXN-D-LACTATE/DEMETHYLMENAQUINONE', 'RXN-

		5642', '2.7.7.57-RXN', 'RXN-5643', 'RXN4FS-3', 'RXN-745', 'RXN-9311', 'RXN1F-28', 'RXN-10708', 'RXN-744', 'NAPHTHOATE-SYN-RXN', 'DMK-RXN', 'RXN-9311', 'O-SUCCINYLBENZOATE-COA-LIG-RXN', 'NAPHTHOATE-SYN-RXN', 'O-SUCCINYLBENZOATE-COA-SYN-RXN'

Appendix B Supplementary material

The predicted essential reaction based on our analysis in the network are presented in table S3, of which most of this reactions were identified to be found in the gold standards or have been predicted to be essential to the network in previous study.

Predicted essential reactions

Table S3: All predicted essential reactions in the network used in this study.

REACTION	No. of knocked Reaction	Essentiality
SUPEROX-DISMUT-RXN	94	1.27027027
CATAL-RXN	74	1
S-ADENMETSYN-RXN	49	0.662162162
2.5.1.19-RXN	48	0.648648649
CHORISMATE-SYNTHASE-RXN	48	0.648648649
SHIKIMATE-KINASE-RXN	48	0.648648649
ADENOSYLHOMOCYSTEINASE-RXN	39	0.527027027
RXN-1321	31	0.418918919
12-OXOPHYTODIENOATE-REDUCTASE-RXN	30	0.405405405
ALLENE-OXIDE-CYCLASE-RXN	30	0.405405405
RXN1F-19	30	0.405405405
IMPCYCLOHYDROLASE-RXN	27	0.364864865
UDPKIN-RXN	27	0.364864865
2.6.1.18-RXN	26	0.351351351
AICARTRANSFORM-RXN	26	0.351351351
3-HYDROXYPROPIONATE-DEHYDROGENASE-RXN	25	0.337837838
PROPCOASYN-RXN	25	0.337837838
RXN-6383	25	0.337837838
RXN-6384	25	0.337837838
DIAMINOPIMDECARB-RXN	24	0.324324324
DIAMINOPIMEPIM-RXN	24	0.324324324
SUCCDIAMINOPIMDESUCC-RXN	24	0.324324324
SUCCINYLDIAMINOPIMTRANS-RXN	24	0.324324324
TETHYDPICSUCC-RXN	24	0.324324324
RXN-10695	23	0.310810811
RXN-10701	23	0.310810811
RXN-5472	23	0.310810811
RXN-10696	22	0.297297297
RXN-10697	22	0.297297297
RXN-10698	22	0.297297297

RXN-10699	22	0.297297297
RXN-10700	22	0.297297297
RXN-10702	22	0.297297297
RXN-10703	22	0.297297297
RXN-10704	22	0.297297297
RXN-10705	22	0.297297297
RXN-10706	22	0.297297297
RXN-10707	22	0.297297297
RXN-5466	22	0.297297297
RXN-5467	22	0.297297297
RXN-5468	22	0.297297297
RXN-5469	22	0.297297297
RXN-5470	22	0.297297297
RXN-5471	22	0.297297297
R601-RXN-FUM/REDUCED-MENAQUINONE	21	0.283783784
ATPPHOSPHORIBOSYLTRANS-RXN	19	0.256756757
HISTIDINE--TRNA-LIGASE-RXN	19	0.256756757
2.7.4.22-RXN	18	0.243243243
DIHYDROFOLATEREDUCT-RXN	18	0.243243243
GLUTAMIDOTRANS-RXN	18	0.243243243
HISTALDEHYD-RXN	18	0.243243243
HISTAMINOTRANS-RXN	18	0.243243243
HISTCYCLOHYD-RXN	18	0.243243243
HISTIDPHOS-RXN	18	0.243243243
HISTOLDEHYD-RXN	18	0.243243243
HISTPRATPHYD-RXN	18	0.243243243
IMIDPHOSDEHYD-RXN	18	0.243243243
PRIBFAICARPISOM-RXN	18	0.243243243
URIDINEKIN-RXN	17	0.22972973
3-HYDROXYISOBUTYRATE-DEHYDROGENASE-RXN	16	0.216216216
3-HYDROXYISOBUTYRYL-COA-HYDROLASE-RXN	16	0.216216216
MEPROPCOA-FAD-RXN	16	0.216216216
METHYLACYLYLCOA-HYDROXY-RXN	16	0.216216216
RXN-1541	15	0.202702703
PABASYN-RXN	14	0.189189189
ADCLY-RXN	13	0.175675676
NAG1P-URIDYLTRANS-RXN	13	0.175675676
PROTOPORGENOXI-RXN	13	0.175675676
DIOHBUTANONEPSYN-RXN	12	0.162162162
NICONUCADENYLYLTRAN-RXN	12	0.162162162
OHMETHYLBILANESYN-RXN	12	0.162162162
PORPHOBILSYNTH-RXN	12	0.162162162

RXN0-1461	12	0.162162162
RXN-3303	12	0.162162162
UROGENDECARBOX-RXN	12	0.162162162
UROGENIIISYN-RXN	12	0.162162162
ADOMET-DMK-METHYLTRANSFER-RXN	11	0.148648649
ANTHRANSYN-RXN	11	0.148648649
ARGSUCCINLYA-RXN	11	0.148648649
ARGSUCCINSYN-RXN	11	0.148648649
DIHYDROFOLATESYNTH-RXN	11	0.148648649
GLUC1PURIDYLTRANS-RXN	11	0.148648649
GTP-CYCLOHYDRO-I-RXN	11	0.148648649
LUMAZINESYN-RXN	11	0.148648649
TRYPTOPHAN--TRNA-LIGASE-RXN	11	0.148648649
4.3.1.14-RXN	10	0.135135135
ACETYLGLUTKIN-RXN	10	0.135135135
ACETYLORNTRANSAM-RXN	10	0.135135135
DIHYDRONEOPTERIN-MONO-P-DEPHOS-RXN	10	0.135135135
H2NEOPTERINALDOL-RXN	10	0.135135135
H2NEOPTERINP3PYROPHOSPHOHYDRO-RXN	10	0.135135135
H2PTERIDINEPYROPHOSPHOKIN-RXN	10	0.135135135
H2PTEROATESYNTH-RXN	10	0.135135135
IGPSYN-RXN	10	0.135135135
LYSINE-23-AMINOMUTASE-RXN	10	0.135135135
N-ACETYLGLUTPREDUCT-RXN	10	0.135135135
PRAISOM-RXN	10	0.135135135
PRTRANS-RXN	10	0.135135135
RXN0-2381	10	0.135135135
RXN0-2382	10	0.135135135
1.4.1.11-RXN	9	0.121621622
2.7.8.15-RXN	9	0.121621622
BETA-LYSINE-56-AMINOMUTASE-RXN	9	0.121621622
BRANCHED-CHAINAMINOTRANSFERILEU-RXN	9	0.121621622
DTMPKI-RXN	9	0.121621622
MALSYN-RXN	9	0.121621622
METHYLACETOACETYLCOATHIOL-RXN	9	0.121621622
R125-RXN	9	0.121621622
RXN-5464	9	0.121621622
SARCOX-RXN	9	0.121621622
SPONTPRO-RXN	9	0.121621622
1.1.1.178-RXN	8	0.108108108
2.4.1.141-RXN	8	0.108108108
2.4.1.142-RXN	8	0.108108108

2KETO-3METHYLVALERATE-RXN	8	0.108108108
2-MEBUCOA-FAD-RXN	8	0.108108108
AIRS-RXN	8	0.108108108
FGAMSYN-RXN	8	0.108108108
RXN-5462	8	0.108108108
RXN-5463	8	0.108108108
SUCCSEMIALDDEHYDROG-RXN	8	0.108108108
TIGLYLCOA-HYDROXY-RXN	8	0.108108108
2.7.7.15-RXN	7	0.094594595
ASPARTATEKIN-RXN	7	0.094594595
CDPDIGLYSYN-RXN	7	0.094594595
DEPHOSPHOCOAKIN-RXN	7	0.094594595
DIPHOSPHOMEVALONTE-DECARBOXYLASE-RXN	7	0.094594595
DMK-RXN	7	0.094594595
DXS-RXN	7	0.094594595
HYDROXYMETHYLGLUTARYL-COA-SYNTHASE-RXN	7	0.094594595
O-SUCCINYLBENZOATE-COA-SYN-RXN	7	0.094594595
PANTOTHENATE-KIN-RXN	7	0.094594595
RXN0-302	7	0.094594595
RXN-7163	7	0.094594595
RXN-7202	7	0.094594595
THI-P-KIN-RXN	7	0.094594595
TRANSALDOL-RXN	7	0.094594595
1.1.1.34-RXN	6	0.081081081
2.7.1.134-RXN	6	0.081081081
2.7.1.148-RXN	6	0.081081081
2.7.7.60-RXN	6	0.081081081
ASPARTATE-SEMIALDEHYDE-DEHYDROGENASE-RXN	6	0.081081081
DAHPSYN-RXN	6	0.081081081
DXPREDISOM-RXN	6	0.081081081
ETHANOLAMINE-KINASE-RXN	6	0.081081081
FRUCTOKINASE-RXN	6	0.081081081
MEVALONATE-KINASE-RXN	6	0.081081081
NAPHTHOATE-SYN-RXN	6	0.081081081
O-SUCCINYLBENZOATE-COA-LIG-RXN	6	0.081081081
PANTEPADENYLYLTRAN-RXN	6	0.081081081
PHOSPHOMEVALONATE-KINASE-RXN	6	0.081081081
P-PANTOCYSDECARB-RXN	6	0.081081081
P-PANTOCYSLIG-RXN	6	0.081081081
RXN-1685	6	0.081081081
RXN-5781	6	0.081081081
RXN-7141	6	0.081081081

RXN-7203	6	0.081081081
RXN-7204	6	0.081081081
RXN-9311	6	0.081081081
SUCROSE-PHOSPHATE-SYNTHASE-RXN	6	0.081081081
THIAZOLSYN3-RXN	6	0.081081081
UREASE-RXN	6	0.081081081
1-ACYLGLYCEROL-3-P-ACYLTRANSFER-RXN	5	0.067567568
3-DEHYDROQUINATE-DEHYDRATASE-RXN	5	0.067567568
3-DEHYDROQUINATE-SYNTHASE-RXN	5	0.067567568
AICARSYN-RXN	5	0.067567568
CITRAMALATE-LYASE-RXN	5	0.067567568
DEOXYRIBOSE-P-ALD-RXN	5	0.067567568
ENTMULTI-RXN	5	0.067567568
GABATRANSAM-RXN	5	0.067567568
GLUTACONYL-COA-DECARBOXYLASE-RXN	5	0.067567568
GLYCEROL-KIN-RXN	5	0.067567568
GTP-CYCLOHYDRO-II-RXN	5	0.067567568
ISOCHORSYN-RXN	5	0.067567568
METHYLASPARTATE-MUTASE-RXN	5	0.067567568
PHOSPHAGLYPSYN-RXN	5	0.067567568
PYRIMSYN3-RXN	5	0.067567568
RIBOPHOSPHAT-RXN	5	0.067567568
RXN-10462	5	0.067567568
RXN-1461	5	0.067567568
RXN-1884	5	0.067567568
SUCROSE-PHOSPHATASE-RXN	5	0.067567568
THI-P-SYN-RXN	5	0.067567568
THYMIDYLATESYN-RXN	5	0.067567568
2.1.1.103-RXN	4	0.054054054
ASPCARBTRANS-RXN	4	0.054054054
CARDIOLIPSYN-RXN	4	0.054054054
CHOLINE-KINASE-RXN	4	0.054054054
CHORISMATEMUT-RXN	4	0.054054054
DHBDEHYD-RXN	4	0.054054054
D-PPENTOMUT-RXN	4	0.054054054
GPPSYN-RXN	4	0.054054054
ISOCHORMAT-RXN	4	0.054054054
MALONYL-ACPDECARBOX-RXN	4	0.054054054
MANNPISOM-RXN	4	0.054054054
METHYLASPARTATE-AMMONIA-LYASE-RXN	4	0.054054054
NICOTINATEPRIBOSYLTRANS-RXN	4	0.054054054
OHACYL-COA-DEHYDROG-RXN	4	0.054054054

OROTPDECARB-RXN	4	0.054054054
PGPPHOSPHA-RXN	4	0.054054054
PRPPAMIDOTRANS-RXN	4	0.054054054
RIBOFLAVINSYNDEAM-RXN	4	0.054054054
RIBOFLAVINSYNREDUC-RXN	4	0.054054054
RXN0-3901	4	0.054054054
RXN-11213	4	0.054054054
RXN1F-28	4	0.054054054
S-2-METHYLMALATE-DEHYDRATASE-RXN	4	0.054054054
SAICARSYN-RXN	4	0.054054054
SAMDECARB-RXN	4	0.054054054
3-OXOACYL-ACP-REDUCT-RXN	3	0.040540541
3-SULFINYL-PYRUVATE-SPON-RXN	3	0.040540541
ACETATEKIN-RXN	3	0.040540541
ACNEULY-RXN	3	0.040540541
ARGDECARBOX-RXN	3	0.040540541
CHORPYRLY-RXN	3	0.040540541
CREATININE-DEAMINASE-RXN	3	0.040540541
CYSTEINE-DIOXYGENASE-RXN	3	0.040540541
DIHYDROOROT-RXN	3	0.040540541
DUTP-PYROP-RXN	3	0.040540541
ENOYL-ACP-REDUCT-NADH-RXN	3	0.040540541
FADSYN-RXN	3	0.040540541
FPPSYN-RXN	3	0.040540541
GALACTOKIN-RXN	3	0.040540541
GDPMANDEHYDRA-RXN	3	0.040540541
GLUCDEHYDROG-RXN-GLC/UBIQUINONE-9	3	0.040540541
GLUCONOKIN-RXN	3	0.040540541
GLYRIBONUCSYN-RXN	3	0.040540541
HOMOSERKIN-RXN	3	0.040540541
IMP-DEHYDROG-RXN	3	0.040540541
KETOGLUTREDUCT-RXN	3	0.040540541
L-ASPARTATE-OXID-RXN	3	0.040540541
MALONYL-COA-ACP-TRANSACYL-RXN	3	0.040540541
NANE-RXN	3	0.040540541
N-CARBAMOYLSARCOSINE-AMIDASE-RXN	3	0.040540541
NICOTINAMID-RXN	3	0.040540541
OROPRIBTRANS-RXN	3	0.040540541
PHOSMANMUT-RXN	3	0.040540541
QUINOPRIBOTRANS-RXN	3	0.040540541
RIBOFLAVIN-SYN-RXN	3	0.040540541
RXN0-2142	3	0.040540541

RXN0-2145	3	0.040540541
RXN0-3921	3	0.040540541
RXN-1083	3	0.040540541
RXN-1883	3	0.040540541
RXN-3761	3	0.040540541
RXN-5761	3	0.040540541
RXN-5763	3	0.040540541
RXN-745	3	0.040540541
RXN-7609	3	0.040540541
RXN-7682	3	0.040540541
RXN-7913	3	0.040540541
RXN-8889	3	0.040540541
RXN-9514	3	0.040540541
RXN-9518	3	0.040540541
RXN-9524	3	0.040540541
RXN-9532	3	0.040540541
RXN-9536	3	0.040540541
RXN-9540	3	0.040540541
RXN-9633	3	0.040540541
RXN-9635	3	0.040540541
RXN-9657	3	0.040540541
RXN-9658	3	0.040540541
RXN-9659	3	0.040540541
RXN-9661	3	0.040540541
RXN-9662	3	0.040540541
RXN-9663	3	0.040540541
RXNQT-4141	3	0.040540541
THREONINE--TRNA-LIGASE-RXN	3	0.040540541
VINYLACETYL-COA-DELTA-ISOMERASE-RXN	3	0.040540541
1.2.1.9-RXN	2	0.027027027
2PGADEHYDRAT-RXN	2	0.027027027
3.5.2.14-RXN	2	0.027027027
3-HYDROXYDECANOYL-ACP-DEHYDR-RXN	2	0.027027027
3-SULFINOALANINE-AMINOTRANSFERASE-RXN	2	0.027027027
4.2.1.58-RXN	2	0.027027027
4.2.1.59-RXN	2	0.027027027
4.2.1.61-RXN	2	0.027027027
ADENPRIBOSYLTRAN-RXN	2	0.027027027
CITSYN-RXN	2	0.027027027
DIHYDRODIPICSYN-RXN	2	0.027027027
ENOYL-COA-HYDRAT-RXN	2	0.027027027
GALACTURIDYLYLTRANS-RXN	2	0.027027027

GLUCONOLACT-RXN	2	0.027027027
NANK-RXN	2	0.027027027
NITRATE-REDUCTASE-NADH-RXN	2	0.027027027
PHENYLPYRUVATE-DECARBOXYLASE-RXN	2	0.027027027
PHOSPHASERDECARB-RXN	2	0.027027027
PREPHENATEDEHYDROG-RXN	2	0.027027027
PROLINE--TRNA-LIGASE-RXN	2	0.027027027
QUINOLINATE-SYNTHA-RXN	2	0.027027027
RIBOFLAVINKIN-RXN	2	0.027027027
RXN0-2144	2	0.027027027
RXN0-3942	2	0.027027027
RXN-1082	2	0.027027027
RXN-5642	2	0.027027027
RXN-5762	2	0.027027027
RXN-8890	2	0.027027027
RXN-9520	2	0.027027027
RXN-9528	2	0.027027027
RXN-9533	2	0.027027027
RXN-9537	2	0.027027027
RXN-9634	2	0.027027027
THRESYN-RXN	2	0.027027027
TYROSINE--TRNA-LIGASE-RXN	2	0.027027027
1.2.1.25-RXN	1	0.013513514
2.1.3.9-RXN	1	0.013513514
2.3.1.168-RXN	1	0.013513514
2.3.1.43-RXN	1	0.013513514
2.3.1.80-RXN	1	0.013513514
2.6.1.30-RXN	1	0.013513514
3-DEHYDROSPHINGANINE-REDUCTASE-RXN	1	0.013513514
3-OCTAPRENYL-4-OHBENZOATE-DECARBOX-RXN	1	0.013513514
4OHBENZOATE-OCTAPRENYLTRANSFER-RXN	1	0.013513514
6PGLUCONOLACT-RXN	1	0.013513514
ACONITATEDEHYDR-RXN	1	0.013513514
ACONITATEHYDR-RXN	1	0.013513514
ADENYLOSUCCINATE-SYNTHASE-RXN	1	0.013513514
AGMATINE-DEIMINASE-RXN	1	0.013513514
AMINOBUTDEHYDROG-RXN	1	0.013513514
AMPSYN-RXN	1	0.013513514
ASNSYNB-RXN	1	0.013513514
ASPARAGINE--TRNA-LIGASE-RXN	1	0.013513514
BRANCHED-CHAINAMINOTRANSFERVAL-RXN	1	0.013513514
BUTYRATE-KINASE-RXN	1	0.013513514

CREATINASE-RXN	1	0.013513514
CREATININASE-RXN	1	0.013513514
CYTIDEAM-RXN	1	0.013513514
DCDPKIN-RXN	1	0.013513514
DCTP-DEAM-RXN	1	0.013513514
GLU6PDEHYDROG-RXN	1	0.013513514
GLUTAMINESYN-RXN	1	0.013513514
GLUTATHIONE-SYN-RXN	1	0.013513514
GLUTCYSLIG-RXN	1	0.013513514
GLUTKIN-RXN	1	0.013513514
GLUTRNAREDUCT-RXN	1	0.013513514
GLUTSEMIALDEHYDROG-RXN	1	0.013513514
GLYOXIII-RXN	1	0.013513514
GLYOXI-RXN	1	0.013513514
GSAAMINOTRANS-RXN	1	0.013513514
HOMSUCTRAN-RXN	1	0.013513514
KDPGALDOL-RXN	1	0.013513514
LYSDECARBOX-RXN	1	0.013513514
LYSOPHOSPHOLIPASE-RXN	1	0.013513514
MALATE--COA-LIGASE-RXN	1	0.013513514
MALYL-COA-LYASE-RXN	1	0.013513514
N-CARBAMOYLPUTRESCINE-AMIDASE-RXN	1	0.013513514
O-SUCCHOMOSERLYASE-RXN	1	0.013513514
PGLUCONDEHYDRAT-RXN	1	0.013513514
PHOSPHATE-BUTYRYLTRANSFERASE-RXN	1	0.013513514
PHOSPHOLIPASE-A1-RXN	1	0.013513514
PMPOXI-RXN	1	0.013513514
PNKIN-RXN	1	0.013513514
PNPOXI-RXN	1	0.013513514
PUTTRANSAM-RXN	1	0.013513514
PYRAMKIN-RXN	1	0.013513514
PYRIDOXKIN-RXN	1	0.013513514
R10-RXN	1	0.013513514
R12-RXN	1	0.013513514
R222-RXN	1	0.013513514
R223-RXN	1	0.013513514
RXN0-1132	1	0.013513514
RXN0-1133	1	0.013513514
RXN0-1147	1	0.013513514
RXN0-1401	1	0.013513514
RXN0-1402	1	0.013513514
RXN0-5217	1	0.013513514

RXN0-742	1	0.013513514
RXN0-743	1	0.013513514
RXN-34	1	0.013513514
RXN-35	1	0.013513514
RXN-5501	1	0.013513514
RXN-5502	1	0.013513514
RXN-5901	1	0.013513514
RXN-6601	1	0.013513514
RXN-6622	1	0.013513514
RXN-6642	1	0.013513514
RXN-7224	1	0.013513514
RXN-7225	1	0.013513514
RXN-7226	1	0.013513514
RXN-7227	1	0.013513514
RXN-7716	1	0.013513514
RXN-7719	1	0.013513514
RXN-7771	1	0.013513514
RXN-7772	1	0.013513514
RXN-7801	1	0.013513514
RXN-7933	1	0.013513514
SERINE-C-PALMITOYLTRANSFERASE-RXN	1	0.013513514
SPERMIDINESYN-RXN	1	0.013513514
SPERMINE-SYNTHASE-RXN	1	0.013513514
URA-PHOSPH-RXN-DEOXYURIDINE/Pi	1	0.013513514

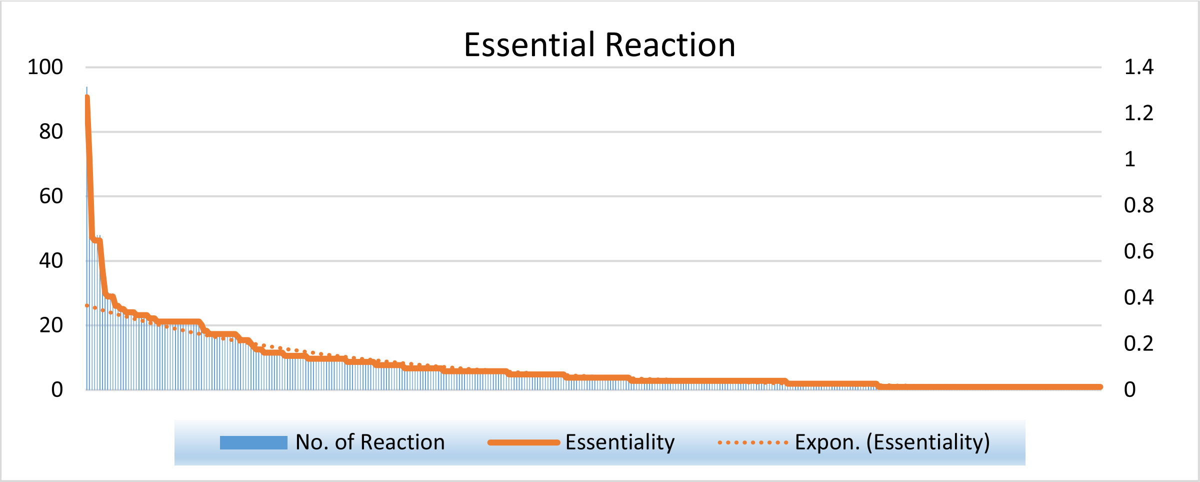

Figure S1: Graph to show No. of reactions considered and essentiality of reactions

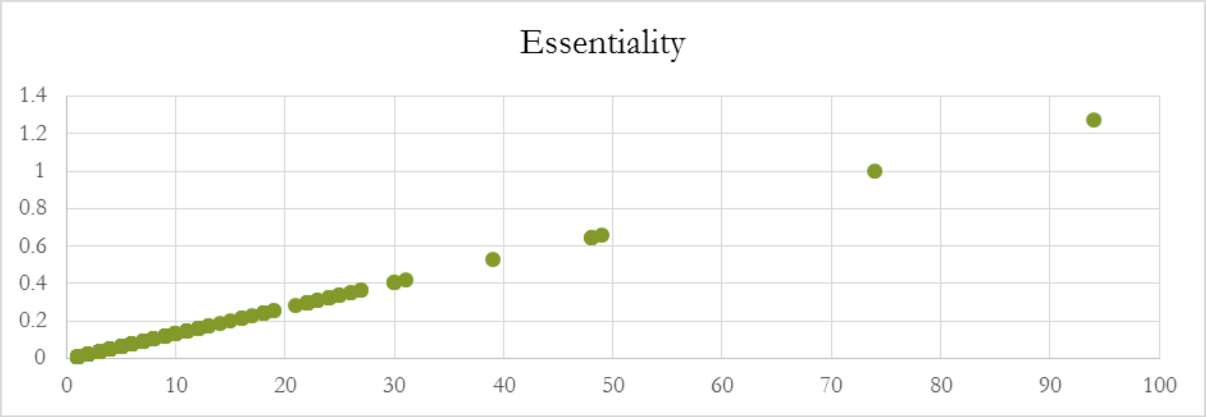

Figure S2: Graph to represent essentiality of reaction

References

[71] S. D. M. Sumanadasa *et al.*, "Antimalarial activity of the anticancer histone deacetylase inhibitor SB939.," *Antimicrob. Agents Chemother.*, vol. 56, no. 7, pp. 3849–56, Jul. 2012.

[72] H. K. Webster and J. M. Whaun, "Antimalarial properties of bredinin. Prediction based on identification of differences in human host-parasite purine metabolism.," *J. Clin. Invest.*, vol. 70, no. 2, pp. 461–9, Aug. 1982.

[73] M. B. Cassera, E. F. Merino, V. J. Peres, E. A. Kimura, G. Wunderlich, and A. M. Katzin, "Effect of fosmidomycin on metabolic and transcript profiles of the methylerythritol phosphate pathway in Plasmodium falciparum," *Mem?rias do Inst. Oswaldo Cruz*, vol. 102, no. 3, pp. 377–384, Jun. 2007.

[74] H. Jomaa *et al.*, "Inhibitors of the Nonmevalonate Pathway of Isoprenoid Biosynthesis as Antimalarial Drugs," *Science (80-.).*, vol. 285, no. 5433, 1999.

[75] A. R. Odom and W. C. Van Voorhis, "Functional genetic analysis of the Plasmodium falciparum deoxyxylulose 5-phosphate reductoisomerase gene," *Mol. Biochem. Parasitol.*, vol. 170, no. 2, pp. 108–111, Apr. 2010.

[76] V. Razakantoanina, N. K. P. Phung, and G. Jaureguiberry, "Antimalarial activity of new gossypol derivatives," *Parasitol. Res.*, vol. 86, no. 8, pp. 665–668, Jul. 2000.

[77] L. Vivas *et al.*, "Plasmodium falciparum: Stage specific effects of a selective inhibitor of lactate dehydrogenase," *Exp. Parasitol.*, vol. 111, no. 2, pp. 105–114, Oct. 2005.

[78] G. A. Biagini *et al.*, "Generation of quinolone antimalarials targeting the Plasmodium falciparum mitochondrial respiratory chain for the treatment and prophylaxis of malaria.," *Proc. Natl. Acad. Sci. U. S. A.*, vol. 109, no. 21, pp. 8298–303, May 2012.

[79] L. Soulère *et al.*, "Screening of Plasmodium falciparum iron superoxide dismutase inhibitors and accuracy of the SOD-assays," *Bioorg. Med. Chem.*, vol. 11, no. 23, pp. 4941–4944, Nov. 2003.

[80] S. Vinayak and Y. D. Sharma, "Inhibition of *Plasmodium falciparum ispH* (*lytB*) Gene Expression by Hammerhead Ribozyme," *Oligonucleotides*, vol. 17, no. 2, pp. 189–200, Jun. 2007.

[81] D. Chakrabarti, S. M. Schuster, and R. Chakrabarti, "Cloning and characterization of subunit genes of ribonucleotide reductase, a cell-cycle-regulated enzyme, from Plasmodium falciparum," *Proc. Natl. Acad. Sci.*, vol. 90, no. 24, pp. 12020–12024, 1993.

[82] J. B. Munro and J. C. Silva, "Ribonucleotide reductase as a target to control apicomplexan diseases," *Curr. Issues Mol. Biol.*, vol. 14, no. 1, pp. 9–26, 2012.

[83] K. S. Lau, A. J. L. Cooper, and D. T. Chuang, "Inhibition of the bovine branched-chain 2-oxo acid dehydrogenase complex and its kinase by arylidenepyruvates," *Biochim. Biophys. Acta - Protein Struct. Mol. Enzymol.*, vol. 1038, no. 3, pp. 360–366, May 1990.

[84] A. M. Vaughan *et al.*, "Type II fatty acid synthesis is essential only for malaria parasite late liver stage development," *Cell. Microbiol.*, vol. 11, no. 3, pp. 506–520, Mar. 2009.

[85] M. Yu *et al.*, "The Fatty Acid Biosynthesis Enzyme FabI Plays a Key Role in the Development of Liver-Stage Malarial Parasites," *Cell Host Microbe*, vol. 4, no. 6, pp. 567–578, Dec. 2008.

[86] J. Baldwin *et al.*, "High-throughput screening for potent and selective inhibitors of Plasmodium falciparum dihydroorotate dehydrogenase.," *J. Biol. Chem.*, vol. 280, no. 23, pp. 21847–53, Jun. 2005.

[87] A. N. Boa, S. P. Canavan, P. R. Hirst, C. Ramsey, A. M. W. Stead, and G. A. McConkey, "Synthesis of brequinar analogue inhibitors of malaria parasite dihydroorotate dehydrogenase," *Bioorg. Med. Chem.*, vol. 13, no. 6, pp. 1945–1967, 2005.

[88] T. Heikkilä *et al.*, "The first de novo designed inhibitors of Plasmodium falciparum dihydroorotate dehydrogenase," 2006.

[89] L. McRobert and G. A. McConkey, "RNA interference (RNAi) inhibits growth of Plasmodium falciparum," *Mol. Biochem. Parasitol.*, vol. 119, no. 2, pp. 273–278, Feb. 2002.

[90] X. Deng *et al.*, “Structural plasticity of malaria dihydroorotate dehydrogenase allows selective binding of diverse chemical scaffolds.,” *J. Biol. Chem.*, vol. 284, no. 39, pp. 26999–7009, Sep. 2009.

[91] R. Gujjar *et al.*, “Lead Optimization of Aryl and Aralkyl Amine-Based Triazolopyrimidine Inhibitors of *Plasmodium falciparum* Dihydroorotate Dehydrogenase with Antimalarial Activity in Mice,” *J. Med. Chem.*, vol. 54, no. 11, pp. 3935–3949, Jun. 2011.

[92] H. J. Painter, J. M. Morrisey, M. W. Mather, and A. B. Vaidya, “Specific role of mitochondrial electron transport in blood-stage Plasmodium falciparum,” *Nature*, vol. 446, no. 7131, pp. 88–91, Mar. 2007.

[93] V. A. Nagaraj, R. Arumugam, D. Prasad, P. N. Rangarajan, and G. Padmanaban, “Protoporphyrinogen IX oxidase from Plasmodium falciparum is anaerobic and is localized to the mitochondrion,” *Mol. Biochem. Parasitol.*, vol. 174, no. 1, pp. 44–52, Nov. 2010.

[94] T. N. C. Ramya, S. Mishra, K. Karmodiya, N. Surolia, and A. Surolia, “Inhibitors of nonhousekeeping functions of the apicoplast defy delayed death in Plasmodium falciparum.,” *Antimicrob. Agents Chemother.*, vol. 51, no. 1, pp. 307–16, Jan. 2007.

[95] N. Suraveratum, S. R. Krungkrai, P. Leangaramgul, P. Prapunwattana, and J. Krungkrai, “Purification and characterization of Plasmodium falciparum succinate dehydrogenase,” *Mol. Biochem. Parasitol.*, vol. 105, no. 2, pp. 215–222, Feb. 2000.

[96] J. Le Bras and R. Durand, “The mechanisms of resistance to antimalarial drugs in Plasmodium falciparum,” *Fundam. Clin. Pharmacol.*, vol. 17, no. 2, pp. 147–153, Apr. 2003.

[97] L. Jiang, P. C. Lee, J. White, and P. K. Rathod, “Potent and selective activity of a combination of thymidine and 1843U89, a folate-based thymidylate synthase inhibitor, against Plasmodium falciparum.,” *Antimicrob. Agents Chemother.*, vol. 44, no. 4, pp. 1047–50, Apr. 2000.

[98] K. M. Massimine *et al.*, “Eosin B as a novel antimalarial agent for drug-resistant Plasmodium falciparum.,” *Antimicrob. Agents Chemother.*, vol. 50, no. 9, pp. 3132–41, Sep. 2006.

[99] J. Krungkrai, R. Kanchanarithisak, S. R. Krungkrai, and S. Rochanakij, “Mitochondrial NADH Dehydrogenase from Plasmodium falciparum and Plasmodium berghei,” *Exp. Parasitol.*, vol. 100, no. 1, pp. 54–61, Jan. 2002.

[100] G. A. Biagini, P. Viriyavejakul, P. M. O’neill, P. G. Bray, and S. A. Ward, “Functional characterization and target validation of alternative complex I of Plasmodium falciparum mitochondria.,” *Antimicrob. Agents Chemother.*, vol. 50, no. 5, pp. 1841–51, May 2006.

[101] S. A. Uyemura, S. Luo, M. Vieira, S. N. J. Moreno, and R. Docampo, “Oxidative phosphorylation and rotenone-insensitive malate- and NADH-quinone oxidoreductases in Plasmodium yoelii yoelii mitochondria in situ.,” *J. Biol. Chem.*, vol. 279, no. 1, pp. 385–93, Jan. 2004.

[102] C. Biot, H. Bauer, R. H. Schirmer, and E. Davioud-Charvet, “5-Substituted tetrazoles as bioisosteres of carboxylic acids. Bioisosterism and mechanistic studies on glutathione

reductase inhibitors as antimalarials," *J. Med. Chem.*, vol. 47, no. 24, pp. 5972–5983, 2004.

[103] P. Grellier, A. Marozienė, H. Nivinskas, J. Šarlauskas, A. Aliverti, and N. Čėnas, "Antiplasmodial activity of quinones: Roles of aziridinyl substituents and the inhibition of Plasmodium falciparum glutathione reductase," *Arch. Biochem. Biophys.*, vol. 494, no. 1, pp. 32–39, Feb. 2010.

[104] K. LÜERSEN, R. D. WALTER, and S. MÜLLER, "Plasmodium falciparum-infected red blood cells depend on a functional glutathione de novo synthesis attributable to an enhanced loss of glutathione," *Biochem. J.*, vol. 346, no. 2, 2000.

[105] Z. Ya, E. Hempelmann, and R. H. Schirmer, "Glutathione reductase inhibitors as potential antimalarial drugs," *Biochem. Pharmacol.*, vol. 37, no. 5, pp. 855–860, Mar. 1988.

[106] Z. Krnajski, T.-W. Gilberger, R. D. Walter, A. F. Cowman, and S. Müller, "Thioredoxin reductase is essential for the survival of Plasmodium falciparum erythrocytic stages.," *J. Biol. Chem.*, vol. 277, no. 29, pp. 25970–5, Jul. 2002.

[107] ‡,§ Rudi A. Baron, ‡ Yuri K. Peterson, ‡ James C. Otto, ∥ and Johannes Rudolph, and ‡,∥ Patrick J. Casey*, "Time-Dependent Inhibition of Isoprenylcysteine Carboxyl Methyltransferase by Indole-Based Small Molecules†," 2006.

[108] A. M. Winter-Vann *et al.*, "A small-molecule inhibitor of isoprenylcysteine carboxyl methyltransferase with antitumor activity in cancer cells.," *Proc. Natl. Acad. Sci. U. S. A.*, vol. 102, no. 12, pp. 4336–41, Mar. 2005.

[109] W. H. Witola, K. El Bissati, G. Pessi, C. Xie, P. D. Roepe, and C. Ben Mamoun, "Disruption of the Plasmodium falciparum PfPMT gene results in a complete loss of phosphatidylcholine biosynthesis via the serine-decarboxylase-phosphoethanolamine-methyltransferase pathway and severe growth and survival defects.," *J. Biol. Chem.*, vol. 283, no. 41, pp. 27636–43, Oct. 2008.

[110] E. Nduati, S. Hunt, E. M. Kamau, and A. Nzila, "2,4-diaminopteridine-based compounds as precursors for de novo synthesis of antifolates: a novel class of antimalarials.," *Antimicrob. Agents Chemother.*, vol. 49, no. 9, pp. 3652–7, Sep. 2005.

[111] A. Banerjee, N. Arora, and U. Murty, "Aspartate carbamoyltransferase of Plasmodium falciparum as a potential drug target for designing anti-malarial chemotherapeutic agents," *Med. Chem. Res.*, 2012.

[112] M. B. Cassera *et al.*, "The methylerythritol phosphate pathway is functionally active in all intraerythrocytic stages of Plasmodium falciparum.," *J. Biol. Chem.*, vol. 279, no. 50, pp. 51749–59, Dec. 2004.

[113] H. Ginsburg, "Progress in in silico functional genomics: the malaria Metabolic Pathways database," *Trends Parasitol.*, vol. 22, no. 6, pp. 238–240, Jun. 2006.

[114] O. Nicolas *et al.*, "Pharmacological properties of a new antimalarial bisthiazolium salt, T3, and a corresponding prodrug, TE3.," *Antimicrob. Agents Chemother.*, vol. 49, no. 9, pp. 3631–9, Sep. 2005.

[115] T. C. Santiago, R. Zufferey, R. S. Mehra, R. A. Coleman, and C. Ben Mamoun, "The Plasmodium falciparum PfGatp is an endoplasmic reticulum membrane protein important for the initial step of malarial glycerolipid synthesis.," *J. Biol. Chem.*, vol.

279, no. 10, pp. 9222–32, Mar. 2004.

[116] P. Gerold and R. T. Schwarz, “Biosynthesis of glycosphingolipids de-novo by the human malaria parasite Plasmodium falciparum,” *Mol. Biochem. Parasitol.*, vol. 112, no. 1, pp. 29–37, Jan. 2001.

[117] N. Surolia and G. Padmanaban, “De novo biosynthesis of heme offers a new chemotherapeutic target in the human malarial parasite,” *Biochem. Biophys. Res. Commun.*, vol. 187, no. 2, pp. 744–750, Sep. 1992.

[118] S. VARADHARAJAN, S. DHANASEKARAN, Z. Q. BONDAY, P. N. RANGARAJAN, and G. PADMANABAN, “Involvement of δ-aminolaevulinate synthase encoded by the parasite gene in de novo haem synthesis by Plasmodium falciparum,” *Biochem. J.*, vol. 367, no. 2, 2002.

[119] R. F. Waller *et al.*, “A type II pathway for fatty acid biosynthesis presents drug targets in Plasmodium falciparum.,” *Antimicrob. Agents Chemother.*, vol. 47, no. 1, pp. 297–301, Jan. 2003.

[120] P. J. Lee *et al.*, “Targeting the Fatty Acid Biosynthesis Enzyme, ?-Ketoacyl?Acyl Carrier Protein Synthase III (PfKASIII), in the Identification of Novel Antimalarial Agents,” *J. Med. Chem.*, vol. 52, no. 4, pp. 952–963, Feb. 2009.

[121] R. F. Waller *et al.*, “Nuclear-encoded proteins target to the plastid in Toxoplasma gondii and Plasmodium falciparum.,” *Proc. Natl. Acad. Sci. U. S. A.*, vol. 95, no. 21, pp. 12352–7, Oct. 1998.

[122] G. A. Kicska, P. C. Tyler, G. B. Evans, R. H. Furneaux, V. L. Schramm, and K. Kim, “Purine-less death in Plasmodium falciparum induced by immucillin-H, a transition state analogue of purine nucleoside phosphorylase.,” *J. Biol. Chem.*, vol. 277, no. 5, pp. 3226–31, Feb. 2002.

[123] M. B. Cassera *et al.*, “Plasmodium falciparum Parasites Are Killed by a Transition State Analogue of Purine Nucleoside Phosphorylase in a Primate Animal Model,” *PLoS One*, vol. 6, no. 11, p. e26916, Nov. 2011.

[124] S. R. Krungkrai *et al.*, “Human malaria parasite orotate phosphoribosyltransferase: functional expression, characterization of kinetic reaction mechanism and inhibition profile,” *Mol. Biochem. Parasitol.*, vol. 134, no. 2, pp. 245–255, 2004.

[125] K. K. Seymour, S. D. Lyons, L. Phillips, K. H. Rieckmann, and R. I. Christopherson, “Cytotoxic Effects of Inhibitors of de Novo Pyrimidine Biosynthesis upon Plasmodium falciparum,” *Biochemistry*, vol. 33, no. 17, pp. 5268–5274, May 1994.

[126] H. J. Khamis, P. S. Sarma, and A. K. Mandal, “Allopurinol as an additive to quinine in the treatment of acute complicated falciparum malaria.,” *Am. J. Trop. Med. Hyg.*, vol. 58, no. 4, pp. 454–457, Apr. 1998.

[127] P. A. Dawson, D. A. E. Cochran, B. T. Emmerson, and R. B. Gordon, “Inhibition of Plasmodium falciparum hypoxanthine-guanine phosphoribosyltransferase mRNA by antisense oligodeoxynucleotide sequence,” *Mol. Biochem. Parasitol.*, vol. 60, no. 1, pp. 153–156, Jul. 1993.

[128] J. D. Artz *et al.*, “Molecular characterization of a novel geranylgeranyl pyrophosphate synthase from Plasmodium parasites.,” *J. Biol. Chem.*, vol. 286, no. 5, pp. 3315–22, Feb. 2011.

[129] T. Triglia, J. G. T. Menting, C. Wilson, and A. F. Cowman, “Mutations in dihydropteroate synthase are responsible for sulfone and sulfonamide resistance in Plasmodium falciparum,” *Proc. Natl. Acad. Sci.*, vol. 94, no. 25, pp. 13944–13949, 1997.

[130] N. Haider, M.-L. Eschbach, S. de S. Dias, T.-W. Gilberger, R. D. Walter, and K. Lüersen, “The spermidine synthase of the malaria parasite Plasmodium falciparum: Molecular and biochemical characterisation of the polyamine synthesis enzyme,” *Mol. Biochem. Parasitol.*, vol. 142, no. 2, pp. 224–236, 2005.

[131] K. Fritz-Wolf *et al.*, “X-ray structure of glutathione S-transferase from the malarial parasite Plasmodium falciparum.,” *Proc. Natl. Acad. Sci. U. S. A.*, vol. 100, no. 24, pp. 13821–6, Nov. 2003.

[132] P. Harwaldt, S. Rahlfs, and K. Becker, “Glutathione S-Transferase of the Malarial Parasite Plasmodium falciparum: Characterization of a Potential Drug Target,” *Biol. Chem.*, vol. 383, no. 5, pp. 821–830, Jan. 2002.

[133] E. Liebau *et al.*, “The glutathione S-transferase from Plasmodium falciparum,” *Mol. Biochem. Parasitol.*, vol. 124, no. 1, pp. 85–90, 2002.

[134] M. Perbandt, C. Burmeister, R. D. Walter, C. Betzel, and E. Liebau, “Native and inhibited structure of a Mu class-related glutathione S-transferase from Plasmodium falciparum.,” *J. Biol. Chem.*, vol. 279, no. 2, pp. 1336–42, Jan. 2004.

[135] R. McLeod *et al.*, “Evidence for the shikimate pathway in apicomplexan parasites,” *Nature*, vol. 393, no. 6687, pp. 801–805, Jun. 1998.

[136] D. Chakrabarti *et al.*, “Protein farnesyltransferase and protein prenylation in Plasmodium falciparum.,” *J. Biol. Chem.*, vol. 277, no. 44, pp. 42066–73, Nov. 2002.

[137] S. Specht *et al.*, “The guanylhydrazone CNI-1493: an inhibitor with dual activity against malaria?inhibition of host cell pro-inflammatory cytokine release and parasitic deoxyhypusine synthase,” *Parasitol. Res.*, vol. 102, no. 6, pp. 1177–1184, May 2008.

[138] S. Fletcher *et al.*, “Potent, Plasmodium-Selective Farnesyltransferase Inhibitors That Arrest the Growth of Malaria Parasites: Structure−Activity Relationships of Ethylenediamine-Analogue Scaffolds and Homology Model Validation,” *J. Med. Chem.*, vol. 51, no. 17, pp. 5176–5197, Sep. 2008.

[139] M. L. Ancelin and H. J. Vial, “Quaternary ammonium compounds efficiently inhibit Plasmodium falciparum growth in vitro by impairment of choline transport.,” *Antimicrob. Agents Chemother.*, vol. 29, no. 5, pp. 814–20, May 1986.

[140] V. Choubey *et al.*, “Inhibition of Plasmodium falciparum choline kinase by hexadecyltrimethylammonium bromide: a possible antimalarial mechanism.,” *Antimicrob. Agents Chemother.*, vol. 51, no. 2, pp. 696–706, Feb. 2007.

[141] A. Crooke, A. Diez, P. J. Mason, and J. M. Bautista, “Transient silencing of Plasmodium falciparum bifunctional glucose-6-phosphate dehydrogenase- 6-phosphogluconolactonase,” *FEBS J.*, vol. 273, no. 7, pp. 1537–1546, Apr. 2006.

[142] J. Preuss *et al.*, “Discovery of a Plasmodium falciparum Glucose-6-phosphate Dehydrogenase 6-phosphogluconolactonase Inhibitor (*R* , *Z*)- *N* -((1-Ethylpyrrolidin-2-yl)methyl)-2-(2-fluorobenzylidene)-3-oxo-3,4-dihydro-2 *H* -benzo[*b*][1,4]thiazine-6-carboxamide (ML276) That Reduces Parasite Growth in Vitro,” *J. Med. Chem.*, vol.

55, no. 16, pp. 7262–7272, Aug. 2012.

[143] R. Zidovetzki, I. W. Sherman, L. O'Brien, H. Kunze, and N. P. Gorkova, "Inhibition of Plasmodium falciparum Phospholipase A 2 by Chloroquine, Quinine, and Arteether," *J. Parasitol.*, vol. 79, no. 4, p. 565, Aug. 1993.

[144] R. Ogwan'g, J. Mwangi, G. Gachihi, A. Nwachukwu, C. R. Roberts, and S. K. Martin, "Use of pharmacological agents to implicate a role for phosphoinositide hydrolysis products in malaria gamete formation," *Biochem. Pharmacol.*, vol. 46, no. 9, pp. 1601–1606, Nov. 1993.

[145] K. Hanada *et al.*, "Plasmodium falciparum Phospholipase C Hydrolyzing Sphingomyelin and Lysocholinephospholipids Is a Possible Target for Malaria Chemotherapy," *J. Exp. Med.*, vol. 195, no. 1, 2001.

[146] K. Yuasa *et al.*, "PfPDE1, a novel cGMP-specific phosphodiesterase from the human malaria parasite Plasmodium falciparum," *Biochem. J.*, vol. 392, no. 1, 2005.

[147] C. J. Taylor, L. McRobert, and D. A. Baker, "Disruption of a *Plasmodium falciparum* cyclic nucleotide phosphodiesterase gene causes aberrant gametogenesis," *Mol. Microbiol.*, vol. 69, no. 1, pp. 110–118, Jul. 2008.

[148] Y. Kitade, A. Kozaki, T. Gotoh, T. Miwa, M. Nakanishi, and C. Yatome, "Synthesis of S-adenosyl-L-homocysteine hydrolase inhibitors and their biological activities," *Nucleic Acids Symp. Ser.*, vol. 42, no. 1, pp. 25–26, Nov. 1999.

[149] S. Shuto, N. Minakawa, S. Niizuma, H. S. Kim, Y. Wataya, and A. Matsuda, "New neplanocin analogues. 12. Alternative synthesis and antimalarial effect of (6???R)-6???-C-methylneplanocin A, a potent AdoHcy hydrolase inhibitor," *J. Med. Chem.*, vol. 45, no. 3, pp. 748–751, 2002.

[150] J. M. Bujnicki, S. T. Prigge, D. Caridha, and P. K. Chiang, "Structure, evolution, and inhibitor interaction of S-adenosyl-L-homocysteine hydrolase from Plasmodium falciparum," *Proteins Struct. Funct. Bioinforma.*, vol. 52, no. 4, pp. 624–632, Aug. 2003.

[151] J. Krungkrai, S. R. Krungkrai, and K. Phakanont, "Antimalarial activity of orotate analogs that inhibit dihydroorotase and dihydroorotate dehydrogenase," *Biochem. Pharmacol.*, vol. 43, no. 6, pp. 1295–1301, Mar. 1992.

[152] K. K. Seymour, A. E. T. Yeo, K. H. Rieckmann, and R. I. Christopherson, "dCTP levels are maintained in *Plasmodium falciparum* subjected to pyrimidine deficiency or excess," *Ann. Trop. Med. Parasitol.*, vol. 91, no. 6, pp. 603–609, Sep. 1997.

[153] P. C. Tyler, E. A. Taylor, R. F. G. Fröhlich, and V. L. Schramm, "Synthesis of 5′-methylthio coformycins: Specific inhibitors for malarial adenosine deaminase," *J. Am. Chem. Soc.*, vol. 129, no. 21, pp. 6872–6879, 2007.

[154] M.-C. Ho *et al.*, "Structural and Metabolic Specificity of Methylthiocoformycin for Malarial Adenosine Deaminases," *Biochemistry*, vol. 48, no. 40, pp. 9618–9626, Oct. 2009.

[155] A. GURANOWSKI, E. STARZYSKA, A. G. McLENNAN, J. BARANIAK, and W. J. STEC, "Adenosine-5'-O-phosphorylated and adenosine-5'-O-phosphorothioylated polyols as strong inhibitors of (symmetrical) and (asymmetrical) dinucleoside tetraphosphatases," *Biochem. J.*, vol. 373, no. 2, 2003.

[156] B. J. Berger, "Antimalarial activities of aminooxy compounds.," *Antimicrob. Agents Chemother.*, vol. 44, no. 9, pp. 2540–2, Sep. 2000.

[157] R. Das Gupta *et al.*, "3-Aminooxy-1-aminopropane and derivatives have an antiproliferative effect on cultured Plasmodium falciparum by decreasing intracellular polyamine concentrations.," *Antimicrob. Agents Chemother.*, vol. 49, no. 7, pp. 2857–64, Jul. 2005.

[158] I. B. Muller, R. Das Gupta, K. Liersen, C. Wrenger, and R. D. Walter, "Assessing the polyamine metabolism of Plasmodium falciparum as chemotherapeutic target," *Mol. Biochem. Parasitol.*, vol. 160, no. 1, pp. 1–7, Jul. 2008.

[159] §,‖,⊥ Sudaratana R. Krungkrai *et al.*, "A Novel Enzyme Complex of Orotate Phosphoribosyltransferase and Orotidine 5‘-Monophosphate Decarboxylase in Human Malaria Parasite Plasmodium falciparum: Physical Association, Kinetics, and Inhibition Characterization†,‡," 2005.

[160] H. SCOTT, A. GERO, and W. OSULLIVAN, "In vitro inhibition of Plasmodium falciparum by pyrazofurin, an inhibitor of pyrimidine biosynthesis de novo," *Mol. Biochem. Parasitol.*, vol. 18, no. 1, pp. 3–15, Jan. 1986.

[161] L. Kotra *et al.*, "Inhibition of Orotidine 5-Monophosphate Decarboxylase and Its Therapeutic Potential," *Mini-Reviews Med. Chem.*, vol. 8, no. 3, pp. 239–247, Mar. 2008.

[162] P. S. Wright, T. L. Byers, D. E. Cross-Doersen, P. P. McCann, and A. J. Bitonti, "Irreversible inhibition of S-adenosylmethionine decarboxylase in Plasmodium falciparum-infected erythrocytes: Growth inhibition in vitro," *Biochem. Pharmacol.*, vol. 41, no. 11, pp. 1713–1718, Jun. 1991.

[163] T. N. C. Ramya, N. Surolia, and A. Surolia, "Polyamine synthesis and salvage pathways in the malaria parasite Plasmodium falciparum," *Biochem. Biophys. Res. Commun.*, vol. 348, no. 2, pp. 579–584, 2006.

[164] C. Wanidworanun, R. L. Nagel, and H. L. Shear, "Antisense oligonucleotides targeting malarial aldolase inhibit the asexual erythrocytic stages of Plasmodium falciparum," *Mol. Biochem. Parasitol.*, vol. 102, no. 1, pp. 91–101, Jul. 1999.

[165] S. Reungprapavut, S. R. Krungkrai, and J. Krungkrai, "*Plasmodium falciparum* Carbonic Anhydrase is a Possible Target for Malaria Chemotherapy," *J. Enzyme Inhib. Med. Chem.*, vol. 19, no. 3, pp. 249–256, Jan. 2004.

[166] A. K. Ghosh, I. Coppens, H. Gårdsvoll, M. Ploug, and M. Jacobs-Lorena, "Plasmodium ookinetes coopt mammalian plasminogen to invade the mosquito midgut.," *Proc. Natl. Acad. Sci. U. S. A.*, vol. 108, no. 41, pp. 17153–8, Oct. 2011.

[167] G. Padmanaban, Z. Q. Bonday, S. Dhanasekaran, and P. N. Rangarajan, "Import of host |[delta]|-aminolevulinate dehydratase into the malarial parasite: Identification of a new drug target," *Nat. Med.*, vol. 6, no. 8, pp. 898–903, Aug. 2000.

[168] S. K. Sharma *et al.*, "Identification, characterization, and inhibition of Plasmodium falciparum beta-hydroxyacyl-acyl carrier protein dehydratase (FabZ).," *J. Biol. Chem.*, vol. 278, no. 46, pp. 45661–71, Nov. 2003.

[169] V. Bulusu, B. Srinivasan, M. P. Bopanna, and H. Balaram, "Elucidation of the substrate specificity, kinetic and catalytic mechanism of adenylosuccinate lyase from Plasmodium

falciparum," *Biochim. Biophys. Acta - Proteins Proteomics*, vol. 1794, no. 4, pp. 642–654, Apr. 2009.

[170] P. J. Thornalley, M. Strath, and R. J. M. Wilson, "Antimalarial activity in vitro of the glyoxalase I inhibitor diester, S-p-bromobenzylglutathione diethyl ester," *Biochem. Pharmacol.*, vol. 47, no. 2, pp. 418–420, Jan. 1994.

[171] M. Urscher, S. S. More, R. Alisch, R. Vince, and M. Deponte, "Tight-binding inhibitors efficiently inactivate both reaction centers of monomeric Plasmodium?falciparum glyoxalase?1," *FEBS J.*, vol. 279, no. 14, pp. 2568–2578, Jul. 2012.

[172] C. M. Crane *et al.*, "Fluorescent Inhibitors for IspF, an Enzyme in the Non-Mevalonate Pathway for Isoprenoid Biosynthesis and a Potential Target for Antimalarial Therapy," *Angew. Chemie Int. Ed.*, vol. 45, no. 7, pp. 1069–1074, Feb. 2006.

[173] R. H. Barker, V. Metelev, E. Rapaport, and P. Zamecnik, "Inhibition of Plasmodium falciparum malaria using antisense oligodeoxynucleotides," *Proc. Natl. Acad. Sci.*, vol. 93, no. 1, pp. 514–518, 1996.

[174] A. L. Bodley, J. N. Cumming, and T. A. Shapiro, "Effects of camptothecin, a topoisomerase I inhibitor, on Plasmodium falciparum," *Biochem. Pharmacol.*, vol. 55, no. 5, pp. 709–711, Mar. 1998.

[175] P. Chavalitshewinkoon-Petmitr, G. Pongvilairat, S. Auparakkitanon, and P. Wilairat, "Gametocytocidal activity of pyronaridine and DNA topoisomerase II inhibitors against multidrug-resistant Plasmodium falciparum in vitro," *Parasitol. Int.*, vol. 48, no. 4, pp. 275–280, Feb. 2000.

[176] S. Gamage, N. Tepsiri, and P. Wilairat, "Synthesis and in vitro evaluation of 9-anilino-3, 6-diaminoacridines active against a multidrug-resistant strain of the malaria parasite Plasmodium falciparum," *J. Med.*, 1994.

[177] W. Noonpakdee, J. Pothikasikorn, W. Nimitsantiwong, and P. Wilairat, "Inhibition of Plasmodium falciparum proliferation in vitro by antisense oligodeoxynucleotides against malarial topoisomerase II," *Biochem. Biophys. Res. Commun.*, vol. 302, no. 4, pp. 659–664, Mar. 2003.

[178] B. Ruan *et al.*, "A unique hydrophobic cluster near the active site contributes to differences in borrelidin inhibition among threonyl-tRNA synthetases.," *J. Biol. Chem.*, vol. 280, no. 1, pp. 571–7, Jan. 2005.

[179] S. Corvaisier, V. Bordeau, and B. Felden, "Inhibition of transfer messenger RNA aminoacylation and trans-translation by aminoglycoside antibiotics.," *J. Biol. Chem.*, vol. 278, no. 17, pp. 14788–97, Apr. 2003.

[180] E. Salcedo, J. F. Cortese, C. V. Plowe, P. F. G. Sims, and J. E. Hyde, "A bifunctional dihydrofolate synthetase–folylpolyglutamate synthetase in Plasmodium falciparum identified by functional complementation in yeast and bacteria," *Mol. Biochem. Parasitol.*, vol. 112, no. 2, pp. 239–252, 2001.

[181] S. MEIERJOHANN, R. D. WALTER, and S. MÜLLER, "Regulation of intracellular glutathione levels in erythrocytes infected with chloroquine-sensitive and chloroquine-resistant Plasmodium falciparum," *Biochem. J.*, vol. 368, no. 3, 2002.

[182] D. F. . Platel, F. Mangou, and J. Tribouley-Duret, "Role of glutathione in the detoxification of ferriprotoporphyrin IX in chloroquine resistant Plasmodium berghei,"

Mol. Biochem. Parasitol., vol. 98, no. 2, pp. 215–223, Jan. 1999.

[183] M. V Flores, D. Atkins, D. Wade, W. J. O'Sullivan, and T. S. Stewart, "Inhibition of Plasmodium falciparum proliferation in vitro by ribozymes.," *J. Biol. Chem.*, vol. 272, no. 27, pp. 16940–5, Jul. 1997.

[184] J. Raman, S. Mehrotra, R. P. Anand, and H. Balaram, "Unique kinetic mechanism of Plasmodium falciparum adenylosuccinate synthetase," *Mol. Biochem. Parasitol.*, vol. 138, no. 1, pp. 1–8, Nov. 2004.

[185] G. A. McConkey, "Plasmodium falciparum: Isolation and Characterisation of a Gene Encoding Protozoan GMP Synthase," *Exp. Parasitol.*, vol. 94, no. 1, pp. 23–32, Jan. 2000.

[186] S. Bazzani, A. Hoppe, and H.-G. Holz, "Network-based assessment of the selectivity of metabolic drug targets in Plasmodium falciparum with respect to human liver metabolism," *BMC Syst. Biol.*, vol. 6, 2012.
